# Cellular senescence in musculoskeletal diseases: biological mechanisms and clinical implications

**DOI:** 10.7150/thno.130018

**Published:** 2026-06-17

**Authors:** Bingfei Li, Wanwan Qi, Bin Zhang, Shuo Ma, Wei Zhang, Caihua Zhang, Wei Xiang, Ran Chen, Chuanqing Bai, Jun Fei, Changyue Gao, Zhenhong Ni, Siru Zhou

**Affiliations:** 1 Department of Rehabilitation Medicine, Army Medical Center, Daping Hospital, Army Medical University of PLA, Chongqing, 400042, China.; 2 Bishan Hospital of Chongqing medical university, Bishan Hospital of Chongqing, Chongqing, 402760, China.; 3 Metabolism and Repair, Laboratory for Prevention and Rehabilitation of Training Injuries, State Key Laboratory of Trauma and Chemical Poisoning, Trauma Center, Research Institute of Surgery, Army Medical Center, Daping Hospital, Army Medical University of PLA, Chongqing, 400042, China.; 4 Department of Wound Infection and Drugs, State Key Laboratory of Trauma and Chemical Poisoning, Daping Hospital, Army Medical University, Chongqing, 400042, China.; 5 Department of Oncology, People's Hospital Affiliated to Chongqing Three Gorges Medical College, 27 Guoben Rd, Chongqing, 404100, China.; 6 School of Life Sciences, Westlake University, Hangzhou, 310030, China.; 7 War Trauma Medical Center, State Key Laboratory of Trauma and Chemical Poisoning, Army Medical Center, Daping Hospital, Army Medical University, Chongqing, 400042, China.

**Keywords:** cellular senescence, skeletal muscle, sarcopenia, osteoporosis, fracture, OA, IDD

## Abstract

Cellular senescence is a persistent state of irreversible growth arrest that occurs when cells encounter various stress signals. It is marked by elevated expression of cell cycle inhibitors, dysregulated gene transcription, and secretion of the senescence-associated secretory phenotype (SASP). These senescent features may exert both detrimental and beneficial effects on tissue homeostasis and systemic physiological integrity. In this review, the relevant pathological processes are categorized into three tissue types: skeletal muscle, bone, and cartilaginous tissue. We systematically delineate the mechanisms of cellular senescence underlying seven musculoskeletal diseases, including skeletal muscle injury and regeneration, sarcopenia, osteoporosis, fracture, osteonecrosis of the femoral head (ONFH), osteoarthritis (OA), and intervertebral disc degeneration (IDD), with a particular focus on the heterogeneity of senescent cells across distinct musculoskeletal diseases. On this basis, we further elaborated on relevant mechanisms and senescence-related targets, and analyzed senescence heterogeneity in diverse musculoskeletal tissues, senescence identification and integrated diagnostic approaches. Moreover, we discussed convergent pathways, the dual roles of senescent cells, and the critical evaluation of disease-specific versus common therapeutic vulnerabilities.

## 1. Introduction

Cellular senescence, a fundamental feature of the aging process 1-3, is characterized by an irreversible cell cycle arrest elicited by various stressors, such as oxidative stress, DNA damage, and oncogenic signaling 4, 5. This state is accompanied by distinct morphological and molecular features, such as lysosomal changes, irreversible cell cycle arrest, resistance to apoptosis, nuclear changes, and the secretion of the senescence-associated secretory phenotype (SASP) 1, 2, 4, 6, 7. Accumulating evidence indicates that cellular senescence exhibits marked heterogeneity across diverse physiological and pathological settings 8, 9. While beneficial senescence plays crucial roles in tissue regeneration 10, wound healing 11, and tumor surveillance 12, the pathological buildup of these cells is a key driver of age-related chronic disorders, including atherosclerosis 13, cataract 14, diabetes 15, and cancer 16. This inherent heterogeneity makes it necessary to distinguish physiologically beneficial senescence from pathologically detrimental senescence. Defining this distinction may support the development of targeted therapeutic strategies that selectively identify and clear deleterious senescent cells, thereby attenuating their pathological consequences.

Musculoskeletal diseases affect approximately 1.71 billion people worldwide. These diseases are commonly linked to chronic pain, limited physical function, disability, and other adverse health outcomes 17. The musculoskeletal system consists of skeletal muscle, bone, cartilage, tendons, ligaments, and various connective tissues. Beyond providing structural support and enabling movement, this system contributes to mechanical integrity, metabolic homeostasis, and several physiological regulatory functions 17, 18. Accumulating evidence suggests that aberrant senescent cell accumulation participates in the onset and progression of musculoskeletal diseases 19, 20 and also influences tissue repair and remodeling within the musculoskeletal system 21. A key feature is the presence of exceptionally long-lived cell populations, including osteocytes in bone 22, chondrocytes in cartilage 23, and post-mitotic myofibers in skeletal muscle 24. Owing to their long lifespan and restricted proliferative potential, these cells are exposed for extended periods to genotoxic, oxidative, and metabolic insults. Therefore, stress-induced senescence, rather than classical replicative senescence, may represent the predominant senescence program in these tissues. Moreover, musculoskeletal tissues, including mineralized bone, proteoglycan-rich cartilage, and collagen-dense connective tissues, reside within highly specialized extracellular matrices and are often poorly vascularized. These structural features restrict cell mobility, limit tissue remodeling, and reduce immune surveillance, thereby impairing the efficient elimination of senescent cells. As a result, senescent cells may progressively accumulate locally and exert sustained effects through the SASP, especially compared with more highly vascularized and immune-surveilled tissues. Thus, a comprehensive investigation into the unique roles and underlying mechanisms of cellular senescence in various musculoskeletal diseases will offer novel insights into their diagnosis and therapeutic strategies.

**Figure 1:**
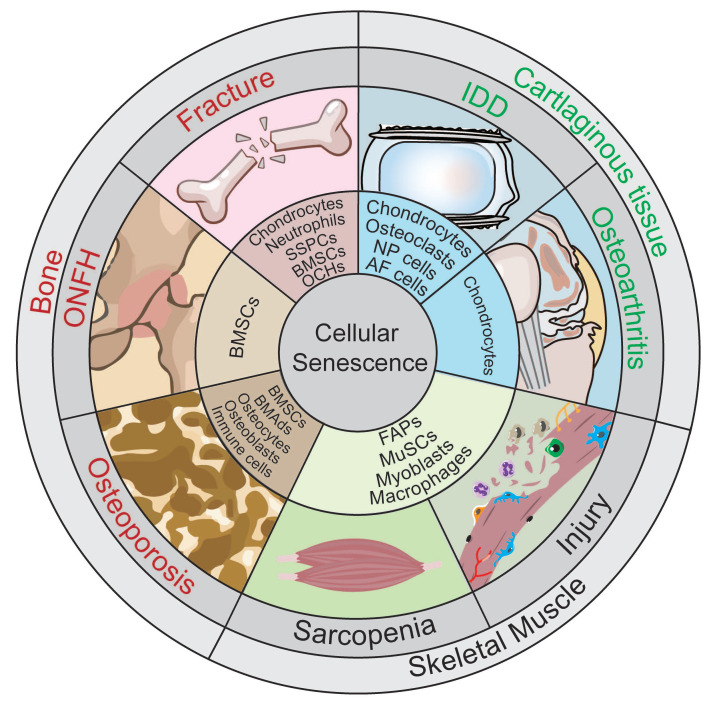
** Senescence of different cell types in musculoskeletal diseases.** Distinct senescent cell types are present in skeletal muscle injury and sarcopenia, including senescent fibro/adipogenic progenitors (FAPs), muscle stem cells (MuSCs), myoblasts, and macrophages. Distinct senescent cell types are involved in different bone diseases. Senescent bone marrow mesenchymal stem cells (BMSCs), osteochondroprogenitors (OCHs), neutrophils, chondrocytes, and skeletal stem/progenitor cells (SSPCs) are observed in fracture; senescent BMSCs, bone-marrow adipocytes (BMAds), osteocytes, osteoblasts, and immune cells are observed in osteoporosis; whereas senescent BMSCs are predominantly observed in osteonecrosis of the femoral head (ONFH). Distinct senescent cell types are present in cartilaginous tissue diseases. Senescent chondrocytes are observed in osteoarthritis (OA); whereas multiple senescent cell types, including nucleus pulposus (NP) cells, annulus fibrosus (AF) cells, cartilage endplate chondrocytes, and osteoclasts, are observed in intervertebral disc degeneration (IDD).

This review highlights the latest advances in cellular senescence research in musculoskeletal diseases. We outline the hallmarks and heterogeneity of senescent cells, current identification approaches, and cell-type specific mechanisms associated with senescence underlying skeletal muscle injury and regeneration, sarcopenia, osteoporosis, fracture, osteonecrosis of the femoral head (ONFH), osteoarthritis (OA), and intervertebral disc degeneration (IDD) **(Figure 1)**. On this basis, we further emphasize precise mechanisms and novel aging targets, and analyze senescence heterogeneity across distinct musculoskeletal tissues, senescence identification methods, and combined diagnostic approaches. Moreover, we discuss convergent pathways, the dual roles of senescent cells, and critically evaluate disease-specific versus common therapeutic vulnerabilities.

## 2. Identifying cellular senescence and their heterogeneity in musculoskeletal tissues

**Figure 2:**
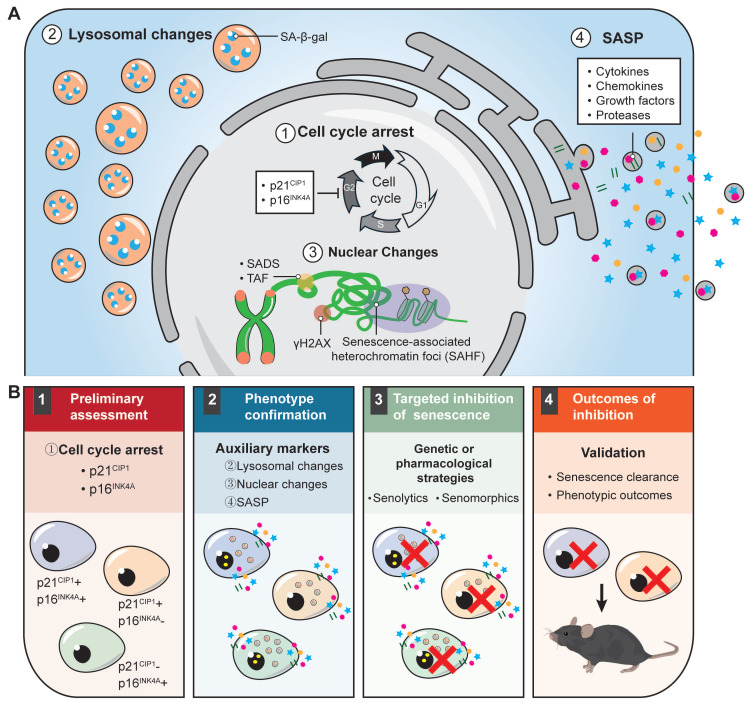
** Hallmarks of senescent cells and a sequential framework for senescence research. (A). ①** Irreversible cell cycle arrest, driven by increased expression of the cyclin-dependent kinase inhibitors p21^CIP1^ and p16^INK4a^, which prevents further cell cycle progression. **②** Lysosomal alterations, indicated by positive staining for senescence-associated β-galactosidase (SA-β-gal). **③** Nuclear alterations, including the formation of senescence-associated heterochromatin foci (SAHF), transposable element-related changes such as senescence-associated distension of satellites (SADS), and the appearance of telomere-associated foci (TAF). They also involve triggering of the DNA damage response, marked by phosphorylation of the histone variant H2AX (γH2AX). **④** The senescence-associated secretory phenotype (SASP) describes the persistent secretion of diverse bioactive molecules by senescent cells, including cytokines, chemokines, growth factors, and proteases. **(B).** 1. Preliminary assessment: Core senescence biomarkers should be screened, particularly the cell-cycle arrest proteins p21^CIP1^ and p16^INK4a^, to identify senescent cell populations. Notably, these populations often display substantial molecular heterogeneity in their expression profiles. 2. Phenotypic confirmation: Senescence status is validated using complementary markers, including lysosomal alterations, nuclear changes, and SASP-related features. 3. Targeted inhibition of senescence: Genetic or pharmacological approaches are applied to modulate senescence, including senolytic agents for the clearance of senescent cells and senomorphic drugs for the suppression of SASP activity. 4. Outcome assessment: The efficacy of senescence inhibition is evaluated by confirming senescent cell clearance and associated phenotypic changes, followed by functional validation in preclinical animal models to determine the pathological or physiological benefits of the intervention.

Although the phenotypic features of senescent cells have been well characterized, no single biomarker can reliably identify cellular senescence, thereby necessitating combined detection approaches 4. However, the optimal selection, number, and combination of senescence markers for sensitive and specific detection remain unresolved. To address this gap, recent guidelines have proposed minimum criteria for senescence studies, including assessment of p21^Cip1^ and p16^Ink4a^ expression, confirmation using auxiliary markers, targeted reduction of senescent cells, and validation of both senescent-cell clearance and phenotypic outcomes 25 **(Figure 2)**. Here, we concentrate on unique considerations for identifying cellular senescence and its heterogeneity in musculoskeletal tissues.

### 2.1 Detection of cell cycle arrest

A durable withdrawal from the cell cycle progression is a defining hallmark of the senescent phenotype. Among the cyclin-dependent kinase inhibitors mediating this cell cycle arrest, p21^Cip1^ and p16^Ink4a^ are the most frequently upregulated ones across diverse senescence models and are therefore widely used as canonical markers of senescent cells 26, 27. During aging in the bone microenvironment, p16^Ink4a^ expression rises markedly across multiple cell populations in both female and male mice, whereas age-related induction of p21^Cip1^ is largely restricted to osteocyte-enriched cells in males. In contrast, analyses of bone-derived cells from young and old women revealed concurrent age-associated increases in both p16^Ink4a^ and p21^Cip1^ expression 28. In another study, aging was associated with selective upregulation of p21^Cip1^, but not p16^Ink4a^, accompanied by DNA damage features and acquisition of a SASP phenotype in a distinct Osx1-Cre labeled osteoprogenitor population 29. Moreover, clearance of p21^Cip1^-positive senescent cells, but not p16^Ink4a^-positive cells, was shown to reduce bone loss and marrow adiposity after radiation-induced skeletal injury 30. This may indicate that p16^Ink4a^ and p21^Cip1^ are involved in senescence in different skeletal cell types within mixed cell populations from biopsy samples. In parallel, p16^Ink4a^ is still difficult to detect reliably in practice and requires strict positive and negative controls 25. For this reason, it is important to further define the distinct roles of p16^Ink4a^ and p21^Cip1^ in senescence-associated musculoskeletal diseases.

### 2.2 Verification of the auxiliary markers of senescent cells

Although p21^Cip1^ and p16^Ink4a^ are among the most commonly used markers of cellular senescence in mammalian systems, their expression alone is not specific enough to define the senescent state. A more thorough evaluation is necessary. In addition to commonly used markers, additional hallmark features of cellular senescence should be assessed, including lysosomal dysfunction, nuclear alterations, and characteristic SASP profiles, which can be examined using histological analyses, biochemical assays, flow cytometry, and other complementary methods.

#### 2.2.1 Lysosomal changes

Lysosomal dysfunction in senescent cells is commonly assessed by senescence-associated β-galactosidase (SA-β-gal) activity at pH 6.0, which arises from increased lysosomal β-galactosidase-mediated hydrolysis of β-D-galactosides 31. SA-β-gal staining is applicable to a wide range of tissues when β-galactosidase activity is preserved, although fresh or freshly frozen specimens are generally preferred for optimal detection. In bone and joint tissues, reliable SA-β-gal detection requires stringent pH control and ethylenediaminetetraacetic acid (EDTA)-based decalcification, as acidic decalcifying agents markedly impair β-galactosidase activity and may lead to false-negative staining. In addition, lysosomal β-D-galactosidase is transcribed and translated from the Glb1 gene 32, suggesting that Glb1 expression can serve as a promising* in vivo* marker for cellular senescence and related tissue dysfunction. To enable dynamic monitoring of aging, a Glb1^+/m^ reporter allele (Glb1-2A-mCherry; GAC) mice has recently been generated, allowing live imaging and lineage tracing of senescent cells at tissues 33. Such reporter mouse models can be combined with two-photon microscopy to enable longitudinal, spatially resolved visualization of senescent cells in the musculoskeletal system in vivo, facilitating the monitoring of senescence-associated changes at both the cellular and tissue levels 34. It is important to note that certain musculoskeletal cell types, such as synovial macrophages in OA 35 and bone-resident osteoclasts 36, exhibit high endogenous β-galactosidase activity owing to their expanded lysosomal compartment. Consequently, the reliability of SA-β-gal as a senescence marker in cells of the monocyte/macrophage lineage remains uncertain and should therefore be complemented by additional senescence markers.

#### 2.2.2 Nuclear alterations

Senescent cells frequently exhibit various nuclear alterations, including nuclear envelope disruption, DNA damage, and senescence-associated distension of satellites (SADS). These changes are not uniform. Instead, they are highly heterogeneous and can differ at the levels of nuclear morphology, molecular features, and functional regulation 37. In terms of morphology, senescent cells usually have enlarged nuclei, a more irregular nuclear shape, enlarged or fragmented nucleoli, and reduced heterochromatin, among other characteristics 38. However, these changes do not occur at the same time in all senescent cells. For example, senescence triggered by oncogenic signaling or DNA replication stress is often accompanied by senescence-associated heterochromatin foci (SAHF), which are highly condensed heterochromatic structures that can be detected by DAPI staining and are enriched with repressive proteins such as HP1. These structures contribute to irreversible growth arrest by suppressing genes that promote proliferation. By contrast, SAHF are rarely seen in replicative senescence or senescence induced by oxidative stress 39.

γH2AX, the phosphorylated form of histone H2AX, is one of the most widely used and reliable markers of DNA damage response (DDR) activation in senescence research. Evidence indicates that the proportion of γH2AX-positive cells in human skeletal muscle does not increase significantly with chronological aging but is markedly elevated in obesity. In particular, irreversibly differentiated postmitotic myonuclei from obese individuals display higher γH2AX levels than those from lean individuals 40. Taken together, these findings suggest that the heterogeneity of nuclear alterations observed in senescent cells reflects differences in cellular identity, senescence-inducing stimuli (e.g., chronological aging, oncogene activation, radiation, and oxidative stress), as well as the surrounding microenvironment.

#### 2.2.3 SASP

SASP is a hallmark of senescent cells. It comprises the stress-induced secretion of a variety of bioactive molecules, including pro-inflammatory cytokines, chemokines, growth factors, and proteases 5. This phenotype exerts a dual regulatory role. Under physiological conditions, it supports tissue repair and immune surveillance, as seen in processes such as fracture healing 41. Conversely, persistent SASP activity triggers chronic inflammation, microenvironmental disruption and tissue dysfunction, thus contributing to skeletal disorders including OA and osteoporosis 42. It should be noted that the SASP is not a static or uniform program. Instead, it exhibits substantial heterogeneity, with its composition and biological functions being shaped by cell type, senescence-inducing stimuli, and temporal dynamics. Research on the role of the SASP in musculoskeletal diseases, including OA and osteoporosis, remains in its infancy. Recently, Saul *et al*. developed SenMayo, a 125-gene senescence signature, and demonstrated that it was enriched in human bone biopsies and could be used to identify senescent cells in mouse models. Further analysis of single-cell RNA sequencing (scRNA-seq) data showed that SenMayo could identify senescent hematopoietic and mesenchymal cells in human and mouse bone marrow at single-cell resolution 43. Therefore, combining SenMayo with single-cell multi-omics, spatial transcriptomics, and proteomics may allow a more precise characterization of SASP heterogeneity and its interactions with the local microenvironment. Distinguishing beneficial from harmful SASP effects may also help develop therapies that regulate SASP rather than directly removing senescent cells, thereby supporting tissue repair while reducing inflammation and degeneration in musculoskeletal diseases.

## 3. Cellular senescence in skeletal muscle

Skeletal muscle, which constitutes approximately 40% of total body weight, is predominant tissue in the human body. Skeletal muscles attach to bones via tendons, and this musculoskeletal system is responsible for facilitating body movements. In addition, skeletal muscles exert a pivotal role in regulating body temperature and maintaining metabolic homeostasis 44. The composition of skeletal muscle involves several cell types, including multinucleated myofibers, muscle stem cells (MuSCs; also known as satellite cells), fibro/adipogenic progenitors (FAPs), endothelial cells, pericyte, macrophages, T cells, and neutrophils 45, 46. Multiple factors, such as aging, genetics, trauma, immobilization, radiation, and medication, can induce pathological changes in skeletal muscle, resulting in a decline in muscle mass and function 47, 48.

In recent years, increasing evidence has shown that senescent cells are present in skeletal muscle and are closely associated with muscle injury and sarcopenia 34, 49-52. This section discusses the dual roles of senescent cells in skeletal muscle regeneration, focusing on their functions and potential mechanisms during muscle injury and sarcopenia, as well as disease-specific features and shared therapeutic targets.

### 3.1 Skeletal muscle injury and regeneration

Muscle injury caused by exercise, trauma, cardiotoxin (CTX), or barium chloride is usually accompanied by myofiber necrosis and initiates a coordinated regenerative response. Following injury, MuSCs become activated, subsequently proliferate, differentiate, and fuse to form new myofibers 53, 54. MuSC activation and myogenic progression are regulated by several transcription factors, which act at distinct stages 55. In their quiescent state, MuSCs maintain stem cell identity through PAX7 expression. Upon activation, these cells begin to express MyoD and MYF5, which promote cell proliferation and commitment to the myogenic lineage. During differentiation, PAX7 expression declines, while myogenin and other myogenic factors increase, facilitating the completion of differentiation and the formation of new myofibers 56.

**Figure 3:**
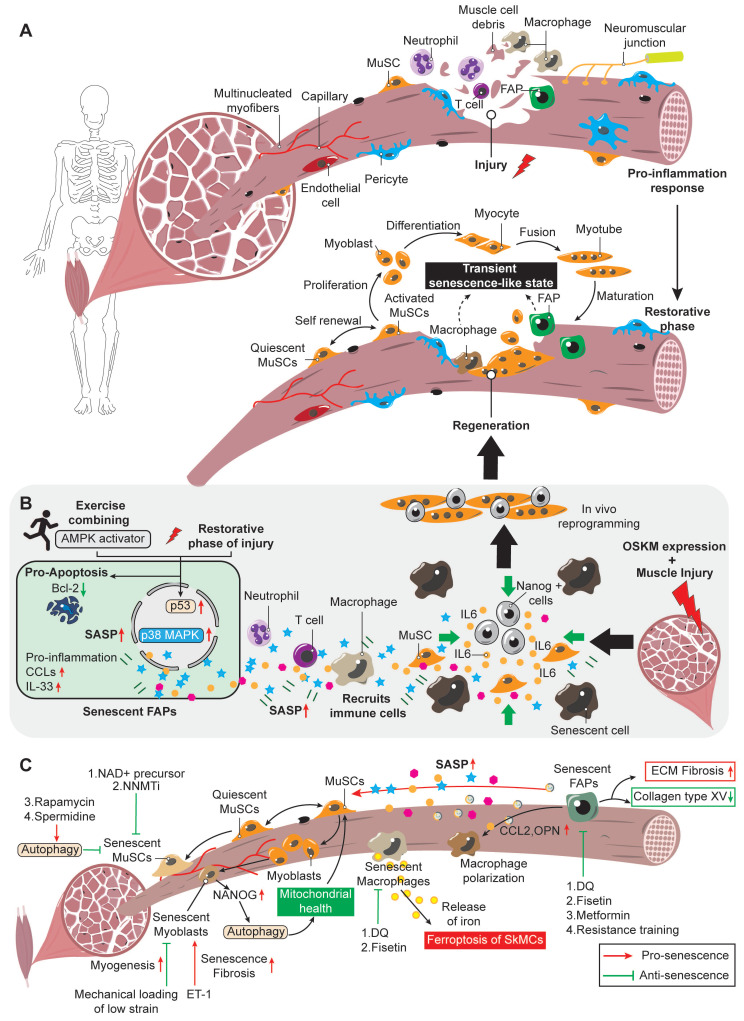
** Cellular senescence in skeletal muscle injury and sarcopenia. (A).** Pro-inflammatory response: Following muscle injury, a pro-inflammatory cascade is initiated. Neutrophils and macrophages infiltrate the injury site to clear myofiber debris. Key cellular players include muscle stem cells (MuSCs), fibro/adipogenic progenitors (FAPs), pericytes, T cells, and endothelial cells. Restorative phase: Quiescent MuSCs become activated and undergo self-renewal and proliferation to generate myoblasts. Myoblasts subsequently differentiate into myocytes, which fuse to form myotubes and eventually mature into new myofibers. FAPs and macrophages also participate in this phase, and a transient senescence-like state may emerge during the regenerative process. **(B).** Exercise combined with AMP-activated protein kinase (AMPK) activation provokes cellular senescence in FAPs. Senescent FAPs are upregulated via the p53 and p38 MAPK signaling pathways and display a senescence-associated secretory phenotype (SASP), typified by high expression of pro-inflammatory factors including CC chemokine ligands and interleukin-33 (IL-33). These SASP factors recruit immune cells and generate a regenerative inflammatory microenvironment favorable for tissue repair. Meanwhile, the expression of the anti-apoptotic protein B-cell lymphoma-2 (Bcl-2) is downregulated in senescent FAPs, rendering them more susceptible to clearance and thereby promoting muscle regeneration. In addition, skeletal muscle injury combined with OSKM (Oct4, Sox2, Klf4, c-Myc) expression can induce cellular senescence in the local tissue. These senescent cells then secrete a range of SASP factors, with IL-6 serving as a key mediator that acts on MuSCs. This paracrine signaling promotes the generation of Nanog+ reprogrammed cells, which have greater stemness and regenerative capacity, ultimately facilitating in vivo lineage reprogramming and improving skeletal muscle repair and regeneration. **(C).** Diagram showing the regulatory network of cellular senescence and related intervention strategies in sarcopenia. After skeletal muscle injury, MuSCs drive a stepwise regenerative process: quiescent MuSCs are activated, proliferate to form myoblasts, and finally fuse into new myofibers. By contrast, pathological senescence disrupts this balance. NAD+ precursors, nicotinamide N-methyltransferase inhibitors (NNMTi), and autophagy activators, including rapamycin and spermidine, can counteract senescence in MuSCs. Endothelin-1 (ET-1) triggers cellular senescence in myoblasts and promotes fibrosis, while low-strain mechanical loading effectively targets senescent myoblasts, enhances myogenesis, and counteracts senescence. Overexpression of the transcription factor NANOG in senescent myoblasts enhances autophagic flux, restores mitochondrial health, and replenishes the Pax7-positive MuSC pool, thereby promoting skeletal muscle regeneration. Senescent macrophages release iron to elicit ferroptosis in skeletal muscle cells (SkMCs) and exacerbate tissue damage, which can be alleviated by eliminating these cells with senolytics such as dasatinib plus quercetin (D+Q) and fisetin. Senescent FAPs secrete SASP factors that impair the function of MuSCs. Among these, pro-fibrotic factors including C-C motif chemokine ligand 2 (CCL2) and osteopontin (OPN) regulate macrophage polarization. In addition, senescent FAPs drive extracellular matrix (ECM) fibrosis and cause collagen type XV depletion. Senolytics (D+Q, fisetin), metformin, and resistance training effectively eliminate senescent FAPs. Red upward arrows indicate upregulation, and green downward arrows indicate downregulation.

The early phase of muscle regeneration is called the pro-inflammatory stage. During this stage, immune cells such as neutrophils 57, macrophages 58, 59, and T cells 59, 60 enter the tissue. In this process, the transition of macrophage phenotype is crucial for muscle regeneration after acute or chronic muscle injury 59. In addition, T cells enter injured muscle and release various cytokines that reshape the local microenvironment, thereby supporting muscle regeneration 60, 61. FAPs within the muscle stem cell niche also serve critical functions in repairing injured muscle. As multipotent progenitors in skeletal muscle, FAPs are capable of differentiating into adipocytes and fibroblasts 62, 63. Beyond this differentiation capacity, FAPs support muscle regeneration by secreting a range of paracrine factors **(Figure 3A)**. A study has shown that aging selectively disrupts MuSC function by reducing the secretion of the matricellular protein WISP1 from FAPs, underscoring the important role of FAPs in establishing a regeneration-supportive microenvironment 64. Moreover, FAP-depleted mice exhibited reduced injury-induced expansion of MuSCs and CD45-positive hematopoietic cells, accompanied by impaired skeletal muscle regeneration 65.

Senescent cells are major contributors to tissue degeneration and age-related diseases due to their permanent cell cycle arrest and the secretion of a pro-inflammatory, pro-fibrotic SASP. Yet within the regenerative microenvironment formed after acute injury, senescent cells may acquire functions that are distinct from their pathological roles and may even support tissue repair.

Senescent cells contribute to tissue degeneration and age-related diseases due to their permanent cell cycle arrest and the secretion of a pro-inflammatory, pro-fibrotic SASP. However, within the regenerative microenvironment that emerges after acute injury, senescent cells may acquire functions distinct from their pathological roles and could even support tissue repair 66. Here, we examine this functional duality of senescent cells in skeletal muscle injury and regeneration.

#### 3.1.1 Beneficial effects of cellular senescence in muscle regeneration

Cellular senescence has traditionally been associated with tissue aging and dysfunction, but accumulating evidence suggests that it may also exert beneficial effects during muscle regeneration. Following acute injury, multiple cell types, including macrophages and FAPs, enter a transient, reversible senescence-like state, as indicated by the upregulation of SASP-related genes **(Figure 3A)**. Their secretory phenotype coordinates immune responses and supports stem cell function, thereby facilitating tissue repair. Early elimination of these cells using senolytics, such as Navitoclax (ABT-263), reduces MuSC numbers and impairs myofiber growth, highlighting that the acute senescence response is important for effective muscle regeneration 67. The underlying mechanism may involve the transient SASP, which provides factors required for MuSC activation and proliferation as well as angiogenesis. Exercise-induced FAPs senescence represents another example of the context-dependent beneficial effects of senescence during muscle regeneration. This study shows that exercise-induced muscle injury promotes FAP senescence in regenerating muscle and helps establish a regenerative inflammatory milieu. In a model of chronic inflammatory myopathy, however, exercise alone fails to promote FAP senescence or overcome their resistance to TNF-α-mediated apoptosis. By contrast, the combination of exercise and pharmacological AMPK activation effectively promotes FAPs senescence and improves muscle regeneration and functional recovery 68. These findings suggest that appropriately induced FAP senescence contributes to muscle homeostasis, whereas impaired induction of this response may be associated with muscle degeneration. Moreover, Chiche *et al*. showed that, following CTX-induced muscle injury and subsequent induction of OSKM (Oct4, Sox2, Klf4, and c-Myc) expression, NANOG-positive (NANOG+) reprogrammed cells were detected at the injury site. These NANOG+ cells were frequently located in close proximity to SA-β-gal-positive senescent cells, and the numbers of these two cell populations were strongly correlated 69. Collectively, these findings suggest that injury-induced cellular senescence establishes a pro-reprogramming microenvironment through the SASP, with interleukin-6 (IL-6) acting as an important contributing factor, thereby promoting cellular plasticity and reprogramming during muscle regeneration **(Figure 3B)**. In regeneration-competent salamanders such as newts, exogenously introduced senescent cells have been shown to enhance the dedifferentiation of mature muscle tissue through paracrine signaling. This effect appears to be mediated, at least in part, by senescence-derived secreted factors acting through the fibroblast growth factor (FGF)-ERK signaling axis, thereby supporting the generation of muscle-derived regenerative progenitors 70. In summary, transient senescence after acute injury can support muscle regeneration by shaping a pro-regenerative microenvironment. Through SASP-mediated paracrine signaling, senescent cells help coordinate immune responses, support MuSCs activity, and promote cellular plasticity. These findings indicate that cellular senescence, when properly induced and resolved, can exert beneficial effects on muscle regeneration.

#### 3.1.2 Detrimental effects of cellular senescence in muscle regeneration

However, when senescent cells persist due to inefficient clearance, sustained SASP production may disrupt the regenerative microenvironment and contribute to tissue dysfunction. In geriatric muscle, MuSCs lose epigenetic repression at the p16^Ink4a^ locus, resulting in p16^Ink4a^ upregulation and a transition from reversible quiescence to an irreversible pre-senescent state. This defect is largely cell-autonomous, as the impaired regenerative capacity of geriatric MuSCs is not restored simply by transplantation into a young host environment 71. Chronic muscle disease further illustrates the detrimental consequences of persistent senescence. In the mdx mouse model of Duchenne muscular dystrophy, repeated cycles of muscle damage and repair are accompanied by sustained accumulation of senescent cells, which is associated with increased fibrosis, inflammation, and muscle weakness 72. The improvement in muscle regeneration after senolytic-mediated reduction of SA-β-Gal-positive cell burden further supports the view that persistent senescence can compromise muscle repair in aged muscle 73.

Further supporting a detrimental role of senescence within the regenerative niche, Moiseeva *et al*. showed that senescent cells emerge in injured skeletal muscle of both young and old mice, while being largely absent from uninjured muscle. These injury-induced senescent cells were predominantly composed of myeloid cells, mainly monocytes and macrophages, FAPs, and MuSCs or their progeny, with a stronger and more persistent accumulation observed in aged muscle 21. Functional experiments further demonstrated that reducing senescent cell burden improved regeneration, whereas transplantation of senescent cells delayed myofiber regeneration. Importantly, senescent cells were deleterious not only when transiently induced after mild injury, but also when persistently accumulated in chronically damaged mdx muscle, indicating that senescence within the muscle niche can compromise regeneration across injury contexts and ages 21. A recent study reported that senescent FAPs may promote macrophage recruitment, favor M2-like macrophage polarization, and reshape FAPs-macrophages communication in aged skeletal muscle 74. These FAPs may alter the local immune environment by secreting factors such as C-C motif chemokine ligand 2 (CCL2) and osteopontin (OPN). Senescent FAPs have distinct secretory profiles. Their secreted factors may increase macrophage infiltration and affect macrophage activation, which can disturb skeletal muscle homeostasis.

At the molecular level, this detrimental functional shift is driven by the sustained activation of several senescence-related signaling pathways. In addition to the canonical p16^Ink4a^-Rb pathway, abnormal and persistent activation of the p53-p21^Cip1^ axis is also critically involved. For example, loss of the endocytic adaptor protein Numb specifically triggers a stable, p53-dependent senescence program in myogenic cells, which differs from the transient and reversible senescence seen in other cell types 51. Similarly, during muscle regeneration, upregulation of the heat shock protein Hsp90β is pivotal for timely shutting down the p53-p21^Cip1^ axis and preventing myoblasts from entering irreversible senescence 75. Inactivation of Hsp90β increases the stability of p53 and causes persistently high expression of p21^Cip1^. Under these conditions, myoblasts become locked in a senescent state and fail to undergo normal proliferation and fusion, thereby directly impairing muscle regeneration. This persistent senescent condition leads to severe regenerative defects, which can be fully rescued by p53 ablation 51. In addition, dysregulation of the p16^Ink4a^ pathway, another central pathway in cellular senescence, is controlled by a key transcriptional regulator in MuSCs. The transcription factor *Slug* serves as a repressor of p16^Ink4a^ transcription, maintaining the reversible quiescence of young MuSCs through its high expression. *Slug* levels decline with age. This decline causes persistent activation of p16^Ink4a^, which promotes MuSC senescence and reduces their self-renewal and regenerative ability. Restoring *Slug* expression can suppress p16^Ink4a^ and improve stem cell function in senescent MuSCs 76. These findings suggest that the *Slug*-p16^Ink4a^ pathway plays an important role in regulating MuSC senescence.

Collectively, persistent senescence inhibits regeneration through a multilevel, dynamically evolving mechanism. Persistent senescence triggers irreversible loss of the inherent regenerative capacity of stem cells and induces cellular accumulation due to impaired clearance. Sustained SASP secretion can disrupt the regenerative microenvironment and aggravate disease progression. These insights highlight the prospect of targeting senescent cells or their SASP to restore tissue repair capacity **(Table 1)**.

### 3.2 Sarcopenia

Sarcopenia is a progressive, age-associated skeletal muscle disorder affecting the whole body. It is clinically defined by marked losses in muscle mass, strength and physical function, which raise the risks of falls, fractures, frailty and premature death 77. Pathologically, this condition manifests as skeletal muscle atrophy, with preferential shrinkage and depletion of type II fast-twitch fibers, accompanied by excessive buildup of intramuscular fat and connective tissue 78. Sarcopenia is associated with a progressive decline in the regenerative capacity of MuSCs, which is essential for supporting tissue repair following muscle injury and trauma. Accumulating evidence reveals that myofiber atrophy stems primarily from protein breakdown governed by the autophagy-lysosome and ubiquitin-proteasome pathways. During muscle wasting, multiple E3 ubiquitin ligases, namely Trim63 (MuRF1) and Fbxo32 (atrogin-1/MAFbx), exhibit increased transcription. This molecular change drives substrate protein polyubiquitination and further facilitates proteasome-dependent degradation in skeletal muscle 79.

#### 3.2.1 Cellular senescence in sarcopenia

Cellular senescence critically contributes to the progression of sarcopenia and the impairment of skeletal muscle regenerative capacity. Skeletal muscle is a highly complex tissue made up of multiple cell types. Among these cells, certain stromal cell populations exhibit distinct senescent phenotypes, specific molecular regulatory patterns, and different influences on the local microenvironment. This section reviews recent advances in senescence research in MuSCs, myoblasts, FAPs, and macrophages in the context of muscle atrophy, discusses the related molecular mechanisms, and summarizes potential targeted therapeutic strategies.

##### 1) MuSCs senescence

As a pivotal seed cell in skeletal muscle, MuSCs senescence directly contributes to the impairment of the regrowth capacity of atrophic skeletal muscle 80. Studies have shown that in aged mice, the level of nicotinamide adenine dinucleotide (NAD+) is reduced in senescent MuSCs, resulting in mitochondrial dysfunction and epigenetic alterations. Administration of the NAD+ precursor nicotinamide riboside has been shown to induce the mitochondrial unfolded protein response and upregulate prohibitin signaling, thereby attenuating MuSC senescence and improving stem cell function in aged mice 81. In addition, Neelakantan *et al*. used a small-molecule inhibitor of nicotinamide N-methyltransferase (NNMT), an enzyme linked to impaired NAD+ salvage metabolism in aged skeletal muscle, to enhance MuSC activation and improve post-injury muscle regeneration 82. Despite differing intervention targets, both studies ultimately achieved significant improvements in the function of senescent MuSCs by elevating NAD+ levels, providing diverse options for the development of drugs to treat sarcopenia. As a key regulatory mechanism for maintaining stemness, the dysfunction of autophagy gives rise to proteostatic imbalance, mitochondrial dysfunction and oxidative stress in MuSCs, ultimately leading to an irreversible senescent phenotype 83. Activation of autophagy by pharmacological agents such as rapamycin (RAPA; an mTOR inhibitor) or spermidine can rescue the function of senescent MuSCs and foster muscle regeneration in sarcopenia.

##### 2) Myoblasts senescence

Myoblasts are activated MuSCs, and their proliferative and differentiation capacity determines the efficiency of muscle recovery. A study showed that endothelin-1 (ET-1) induces cellular senescence and fibronectin expression in cultured murine myoblasts through activation of the ET_A_ receptor, with this effect mediated by reactive oxygen species (ROS) generation and the PI3K-AKT-GSK signaling pathway 84. Although the *in vivo* data were correlative, aged mice exhibited higher circulating ET-1 levels, reduced grip strength, increased muscular fibrosis, and elevated p16^Ink4a^ expression, supporting a potential link between ET-1 signaling and age-related skeletal muscle dysfunction. In addition to molecular interventions, mechanical stimuli also appear to influence senescent myogenic cells. The study showed that mechanical loading could promote myogenic differentiation and cell survival in senescent myoblasts, although these effects were markedly weaker than those observed in control cells. Among the tested loading conditions, low-strain mechanical loading at 2% produced the most evident response in senescent myoblasts and more effectively modulated the upregulation of myogenic factors 85. Moreover, genetic manipulation can also reverse myoblast senescence. Overexpression of the transcription factor NANOG in senescent myoblasts can enhance autophagic flux to clear dysfunctional mitochondria, thereby overcoming the effects of cellular senescence. In a mouse model of premature aging, senescent cells expressing NANOG also replenished the pool of Pax7-positive myogenic progenitors and promoted the formation of eMyHC-positive myofibers 86.

##### 3) FAPs senescence

FAPs are important stromal cells that regulate the stem cell regenerative microenvironment, and their senescence is also a key factor driving muscle atrophy. FAPs represent the major senescent cell type in multiple models, including physiological aging 50, 74, Hutchinson-Gilford progeria syndrome (HGPS) 87, and inclusion body myositis (IBM) 88. Senescent FAPs impair muscle recovery through the SASP. In the HGPS model, they inhibit MuSC proliferation and myogenic differentiation through paracrine signaling, thereby compromising stem cell function 87. Senescent FAPs also disturb the extracellular matrix (ECM) by altering collagen composition. In IBM, the expression of collagen type XV, which plays a vital role in the structural integrity of muscle fibers, is lost. The SASP of these senescent cells is abundant in ECM-remodeling proteins, and this profile ultimately gives rise to fibrosis 88. Moreover, a study based on a disuse model induced by hindlimb immobilization or suspension in aged C57BL/6J mice found that muscle disuse can trigger cellular senescence in multiple skeletal muscle cell types, with FAPs being particularly affected. These senescent cells then aggravate inflammation, enhance ECM fibrosis, and ultimately hinder the functional recovery of muscle 89. In addition, senescent FAPs promote the recruitment and phenotypic polarization of immune cells by highly expressing and secreting CCL2 and OPN. Both factors can effectively attract macrophages and drive them toward a pro-fibrotic M2 phenotype 74.

Since senescent FAPs are closely involved in impaired muscle recovery and muscle atrophy, a range of targeted strategies has been developed to limit their adverse effects. These approaches mainly include senolytic elimination, senomorphic regulation, and exercise-related interventions. For instance, the senolytic agent fisetin can selectively clear senescent FAPs, which helps recover MuSCs function and ameliorate pathological changes in muscle in progeroid mouse models 87. Likewise, in aged mice, the combination of dasatinib and quercetin (D+Q) can reverse aging-related molecular and structural alterations and improve skeletal muscle strength 50. In terms of senomorphic regulation, metformin treatment lowers the expression of senescence markers such as SA-β-gal in FAPs from aging skeletal muscle during recovery after disuse atrophy. At the same time, it redirects FAPs fate toward a more adipogenic and less myofibroblast-like phenotype, which contributes to improved ECM remodeling 89. Resistance training, as a non-pharmacological intervention, can also substantially reduce the proportion of senescent FAPs in the skeletal muscle of aged rats and shows benefits comparable to those of senolytic treatment 90. Overall, these pharmacological and physiological strategies that modulate senescent FAPs may attenuate muscle atrophy and support the recovery of skeletal muscle function.

##### 4) Macrophages senescence

Macrophages are an important immune cell population in skeletal muscle and play key roles in tissue homeostasis, injury responses, and regeneration 91. Macrophage senescence is also closely linked to changes in the skeletal muscle microenvironment. ScRNA-seq analysis has revealed dynamic alterations in macrophage subpopulations in aged skeletal muscle, with upregulated expression of pro-inflammatory markers and senescence-associated markers 92. Such changes may form the inflammatory basis underlying muscle atrophy. Our recent work has provided mechanistic evidence that senescent macrophages directly contribute to pathological muscle atrophy 34. We observed that in OA-associated muscle atrophy, infiltrating macrophages display a senescent phenotype, hallmarked by increased p16^Ink4a^ expression, elevated SA-β-gal activity, increased abundance of the DNA damage marker γH2AX, and enhanced expression of SASP factors including multiple proinflammatory cytokines. These senescent macrophages induce ferroptosis in skeletal muscle cells* via* paracrine signaling, thereby accelerating muscle atrophy. Notably, elimination of these senescent cells with the combination of D+Q, or exogenous supplementation of CoQ10, both effectively mitigate muscle atrophy. Furthermore, one study has demonstrated that macrophages in muscle exhibit senescent characteristics and suppress MuSCs function* via* SASP secretion in muscular dystrophy disease mice. Eliminating senescent macrophages with the senolytic fisetin helps recover stem cell counts and improve muscle phenotype 93. These findings confirm that senescent macrophages act as key mediators in multiple distinct models of muscle atrophy.

Of note, the detection of canonical senescence markers, such as p16^Ink4a^ expression and SA-β-gal activity, in macrophages does not necessarily indicate that these cells have entered an irreversible senescent state 94. Evidence from multiple studies indicates that macrophages reversibly express p16^Ink4a^ and SA-β-gal in reaction to physiological immune stimuli, in a manner independent of the p53 pathway 95. This process is fundamentally different from genuine cellular senescence in terms of both how it is induced and whether the phenotype can be reversed. Future therapeutic development therefore requires more accurate identification of pathogenic senescent macrophages and better discrimination between bona fide senescence and reversible senescence-like immune states. Treatment strategies should integrate multiple senescence-related features, such as SASP profiles, DNA damage signaling, and cell-cycle arrest markers, rather than relying on a single marker, to improve specificity and reduce off-target effects. Overall, cellular senescence provides an important framework for understanding skeletal muscle dysfunction and impaired regeneration. A key challenge is to distinguish its context-dependent beneficial and detrimental roles and to selectively target pathogenic senescent cells while preserving or restoring the regenerative capacity of muscle tissue **(Figure 3C)**.

## 4. Cellular senescence in bone

Bone is not merely a rigid scaffold but a vital organ that supports and protects the body. It also stores minerals such as calcium and phosphorus and contributes to hematopoiesis and endocrine regulation. Bone homeostasis depends on continuous remodeling, which is coordinated by osteoblasts responsible for bone formation, osteoclasts responsible for bone resorption, and osteocytes embedded within the bone matrix. This balance can be disrupted in skeletal disorders such as osteoporosis, impaired fracture healing, and osteonecrosis of the femoral head (ONFH). Increasing evidence suggests that pathological accumulation of senescent cells may be an important mechanism involved in these changes.

### 4.1 Osteoporosis

Osteoporosis is a systemic skeletal disorder characterized by reduced bone mass and density, impaired bone microarchitecture, elevated bone fragility, and a higher risk of fracture 96. Osteoporosis is primarily caused by an imbalance between osteoclasts and osteoblasts, resulting in bone resorption exceeding bone formation 97. Farr *et al*. demonstrated that multiple cell types within the bone microenvironment of naturally aged mice exhibit features of cellular senescence, including osteocytes, osteoblast-lineage cells, myeloid cells, B cells, and T cells 98. Notably, senescent osteocytes and myeloid cells appeared to be major contributors to the SASP within the aged bone microenvironment. In old mice with established age-related bone loss, genetic clearance of p16^Ink4a^-positive senescent cells using the *INK-ATTAC* system, pharmacological clearance with the senolytic combination D+Q, or SASP suppression with a JAK inhibitor improved bone mass, bone strength, and bone microarchitecture 98.

#### 4.1.1 Bone marrow mesenchymal stem cells (BMSCs) senescence in osteoporosis

BMSCs are multipotent stem cells localized in the bone marrow that can give rise to adipocytes, chondrocytes, and osteoblasts, and contribute to bone regeneration and remodeling by maintaining bone tissue homeostasis 99. BMSCs senescence directly impairs osteogenic capacity, thereby triggering bone loss and osteoporosis. Based on current studies, BMSCs senescence not only underlies the decline of cell-autonomous response but also serves as a key mediator of systemic dysregulation in the bone microenvironment 100.

BMSCs senescence in osteoporosis is a pathological process triggered by multiple stressors, mediated through specific signaling pathways, and ultimately leading to functional failure. A study has indicated that the accumulation of advanced glycation end products (AGEs) induces excessive ROS generation in BMSCs in a concentration-dependent manner, resulting in mitochondrial dysfunction, a key driver of BMSCs senescence in senile osteoporosis (SOP) 101. Additionally, radiation and chemotherapeutic agents such as doxorubicin provoke DNA damage and initiate a sustained DNA damage response, directly inducing therapy-related senescence in BMSCs and osteocytes 102, 103. In diabetic osteoporosis (DOP), metabolic disturbances such as hyperglycemia are closely linked to dysregulation of the Ezh2-Nrf2 signaling axis, which underlies the increased abundance of senescent leptin receptor-positive BMSCs (LepR+ BMSCs) 104. Subsequently, these stress signals converge on the central senescence effector pathways. Many studies have shown that the p53-p21^Cip1^ and p16^Ink4a^-Rb pathways are markedly activated in senescent BMSCs, leading to irreversible cell cycle arrest 105, 106. Meanwhile, autophagy, especially mitophagy, which is a key process for maintaining intracellular homeostasis, is inhibited. For example, downregulation of sirtuin3 (Sirt3) expression impairs mitophagy and accelerates BMSCs senescence 101. However, loss of function of the autophagy receptor optineurin (OPTN) results in the accumulation of fatty acid-binding protein 3 (FABP3) protein and drives BMSCs senescence 107.

One of the core characteristics of BMSCs senescence is the activation and release of the SASP. Studies have shown that the expression of SASP is co-regulated by multiple signaling pathways, among which the NF-κB and p38 MAPK-MK2 pathways act as key initiators and sustainers 98, 102, 108. For instance, in chemotherapy-induced osteoporosis models, the DNA damage response drives the massive secretion of SASP factors through sustained activation of the p38 MAPK-MK2 axis 102. Meanwhile, aberrant activation of the mTOR signaling pathway, such as LRRc17-mediated inhibition of mitophagy through the PI3K/mTOR axis 105, as well as dysregulation of epigenetic regulators including Ezh2 and ALKBH5, further stabilize and amplify the effects of SASP 109, 110. The composition of the SASP is heterogeneous and influenced by different senescence-inducing factors, such as aging, radiation, estrogen deficiency, and high glucose, which determines its divergent effects on the bone microenvironment 28, 111, 112. Senescent BMSCs and their secreted SASP factors not only trigger a self-amplifying senescence cascade but also remodel the immune microenvironment, thereby acting as a critical bridge linking cellular senescence to immune dysregulation 28, 113. Upregulated secretion of pro-inflammatory cytokines within the SASP effectively activates the receptor activator of nuclear factor-κB ligand (RANKL) signaling pathway and macrophage colony-stimulating factor (M-CSF), closely correlating with osteoclastogenesis and the subsequent increase in bone resorption 114. The chronic low-grade inflammatory environment established by SASP directly promotes the recruitment and polarization of immune cells. For instance, SASP components such as CCL2 and CCL7 mediate the recruitment of monocytes/macrophages to the bone microenvironment 28, 115. More importantly, SASP can propagate the senescent phenotype to neighboring healthy cells in a paracrine manner 116. Meanwhile, BMSC senescence is associated with reduced osteogenic potential and enhanced adipogenic differentiation, which may contribute to impaired bone formation and increased bone marrow adipogenesis 101, 103.

#### 4.1.2 Osteoblast senescence in osteoporosis

Osteoblasts, the primary functional cells mediating bone matrix synthesis and mineralization, play an essential role in maintaining bone microenvironmental homeostasis and bone mass balance 117. Osteoblast senescence has been identified as a critical pathological event that fuels the progressive decline in bone formation and disrupts bone homeostasis 98, 118. It has been shown that in glucocorticoid-induced osteoporosis (GIO), the *Cmpk2* gene directly mediates the senescence process of osteoblasts by regulating mitochondrial dysfunction and impairs their differentiation capacity, revealing that metabolic disturbance is a critical upstream event triggering senescence 119. Similarly, targeted ablation of Men1 induces osteoblast senescence and disturbs the balance between bone formation and resorption in age-related osteoporosis, indicating that loss of specific gene function can independently drive senescence 120. Oxidative stress is another contributor to osteoblast senescence. ROS-induced downregulation of peptidyl arginine deiminase 2 (PADI2) accelerates senescence in MC3T3-E1 cells, an osteoblast-like cell line, by promoting the production of pro-inflammatory SASP factors and activating the NF-κB pathway, ultimately disrupting bone homeostasis and contributing to age-related bone loss 115.

Recent studies have revealed that intervening in osteoblast senescence has become a highly promising therapeutic strategy. Bone morphogenetic protein 9 (BMP9) has been shown to effectively suppress the senescence program of osteoblasts through the Smad1-Stat1-p21^Cip1^ signaling pathway, thereby improving bone microarchitecture and bone mass in aged mice 121. This suggests a physiological role for BMP family members in maintaining the youthful state of osteoblasts. Alternatively, activation of the vitamin D receptor (VDR) attenuates both ferroptosis and senescence in osteoblasts by activating the Nrf2/GPX4 antioxidant pathway, offering a new perspective for intervening in age-related osteoporosis* via* the integrated metabolic-oxidative stress network 122. Overall, targeting osteoblast senescence offers a novel therapeutic strategy to reverse defects in bone formation and achieve fundamental treatment of osteoporosis.

#### 4.1.3 Osteocyte senescence in osteoporosis

Osteocytes are highly active and functionally diverse cells in bone, playing key roles in bone remodeling, mechanosensing, and endocrine regulation 123. They also serve as a crucial source of RANKL, which is required for osteoclastogenesis and acts as a multifunctional cytokine involved in the regulation of bone metabolism and immune homeostasis 123, 124. As post-mitotic cells, osteocytes gradually undergo senescence, which has been recognized as an important cellular mechanism underlying the initiation and progression of age-related osteoporosis 98, 125. Senescent osteocytes lose their normal physiological functions and concomitantly secrete large amounts of pro-inflammatory factors, chemokines and matrix-degrading enzymes. These SASP components form a persistent low-grade inflammatory environment in the bone microenvironment, directly suppressing osteoblast activity and promotes osteoclast production, thus disrupting bone homeostasis 28.

Senescent osteocytes are associated with specific molecular pathways and signaling molecules. Senescence in osteoblast-lineage cells has been shown to increase Tnfsf11/RANKL expression, which may contribute to osteoclast accumulation and age-associated cortical bone resorption. In aged mice, senolytic treatment with ABT-263 reduced senescence markers and decreased Tnfsf11 expression in osteocyte-enriched cortical bone 126. Furthermore, senescent osteocytes may release exosomes with dysregulated microRNA profiles. In male senescence-accelerated mouse prone 6 (SAMP6) mice, exosomes derived from senescent osteocyte-like cells exhibited reduced miR-494-3p expression, which relieved the inhibition of phosphatase and tensin homolog (PTEN), impairing osteoblast differentiation, and accelerating age-related bone loss 127. This inhibitory effect can be reversed by miR-494-3p mimics. Moreover, senescent osteocytes trigger the NF-κB and MAPK inflammatory signaling cascades, thereby suppressing bone formation and enhance bone resorption, and interventions such as RAPA or senolytics can clear these cells to alleviate bone loss 128.

In 20-22-month-old naturally aged mice, oxidative stress and DNA damage induce a senescent osteocyte phenotype characterized by high p16^Ink4a^ expression, elevated SA-β-gal activity, and increased SASP secretion. Bone-targeted delivery of β-galactose-modified maytansine (DM1-Gal) selectively clears these senescent osteocytes and markedly attenuating age-related bone loss 129. Similarly, in SAMP6 mice, senescent cell accumulation increases SA-β-gal activity and pro-inflammatory SASP secretion to inhibit bone formation, while the galactose-modified tetraphenylethylene prodrug (TPE-Gal) is specifically activated by SA-β-gal to generate active TPE-OH, inducing senescent cell apoptosis and improving osteoporosis and bone injury healing 130. Radiation is another trigger. In 2 Gy γ-ray-irradiated osteocyte-like MLO-Y4 cells and whole-body irradiated mice, radiation-induced osteocyte senescence inhibits the osteogenic differentiation potential of BMSCs through paracrine SASP factors, including IL-6 and matrix metalloproteinase 3 (MMP-3), thereby leading to bone loss 103. A study has found that local clearance of senescent osteocytes using the *DMP1-Cre*-driven *p16-LOX-ATTAC* model results in partial improvement of spinal bone mass, with no effect on femoral bone mass 131. Conversely, systemic clearance of senescent cells using the *p16-INK-ATTAC* model achieves more comprehensive improvement in both spinal and femoral bone mass, while simultaneously reducing bone resorption and bone marrow adiposity 131. Collectively, osteocyte senescence contributes to bone loss under multiple triggers, including aging, oxidative stress, and radiation, while its selective elimination may help restore bone remodeling balance and mitigate osteoporosis progression.

#### 4.1.4 Bone-marrow adipocytes (BMAds) senescence in osteoporosis

Bone marrow adipose tissue (BMAT) is a specialized adipose depot. It serves as an essential component of the bone marrow stroma and possesses unique paracrine and endocrine functions 132. BMAds, which are terminally differentiated from BMSCs, are the major cellular component of BMAT 133. Distinct from adipocytes in white adipose tissue and brown adipose tissue in lineage origin, BMAds play crucial regulatory roles in both local and systemic metabolism.

Studies have shown that in GIO, glucocorticoids drive bone loss not by promoting adipogenic differentiation of BMSCs through the conventional mechanism, but by directly inducing senescence in already differentiated BMAds 134, 135. Senescent BMAds induce secondary senescence in bone vascular endothelial cells and osteoblasts through the secretion of inflammatory factors, such as IL-6 and TGF-β, thereby leading to bone loss. Blocking BMAds senescence* via* Adipoq-Cre-mediated p16^Ink4a^ gene knockout or treatment with the perspective on peroxisome proliferator-activated receptor γ (PPARγ) antagonist can alleviate senescence in the bone microenvironment and improve osteoporosis 135. Xie *et al*. demonstrated that in aged mouse models, proliferating cell nuclear antigen (PCNA)-clamp associated factor (PCLAF) secreted by bone marrow macrophages induces senescence of BMAds through ligation to the ADGRL2 receptor 136. These senescent cells then secrete SASP factors, which inhibit osteoblast function and promote osteoclast activity, thereby exacerbating osteoporosis. Kumar *et al*. found that in Alzheimer's disease transgenic mouse models and naturally aged mouse models, senescent BMAds promote amyloid-beta deposition by secreting serum amyloid P component, leading to reduced bone mass and osteoporosis 137. BMAd senescence contributes to the progression of GIO and SOP by reshaping the bone microenvironment, enhancing inflammatory responses, and disturbing the balance between osteogenesis and osteoclastogenesis.

#### 4.1.5 Immune cells senescence in osteoporosis

Immune cells are closely involved in the regulation of bone metabolism 138. Different immune cell populations release cytokines and other signaling molecules that affect both bone formation and bone resorption 139, 140. Studies on age-related bone loss have found senescent immune cells in the bone marrow, including bone marrow macrophages (BMMs) and neutrophils. 141. These cells can disrupt normal bone metabolism through diverse molecular pathways, thereby further promoting the progression of osteoporosis.

One study reported that senescent immune cells release grancalcin. This protein can directly inhibit plexin-B2 signaling. As a result, the osteogenic differentiation of bone-forming precursor cells is impaired, which may further aggravate bone loss. Notably, targeted approaches such as grancalcin-neutralizing antibodies and deletion of the grancalcin gene improved skeletal aging phenotypes and alleviated osteoporosis 141. In addition, Jing *et al*. reported that senescent BMMs release miR-378a-3p-enriched extracellular vesicles (EVs). These EVs can be taken up by target cells. The delivered miR-378a-3p then inhibits PPARα signaling, which is important for bone homeostasis. This process disrupts bone metabolism and promotes bone loss 142. By contrast, fenofibrate, a known PPARα agonist, can reduce the pro-senescent and bone-damaging effects of these EVs. It also helps restore tissue homeostasis, including bone homeostasis, and extends lifespan in experimental models 143.

Taken together, different senescent cells in skeletal tissues secrete SASP factors that inhibit osteogenic differentiation and mineralization, ultimately disrupting bone remodeling and promoting bone loss **(Table 2)**. Studies have shown that selective removal of these senescent cells can reduce bone marrow inflammation and help maintain bone mass and bone strength 144-146.

### 4.2 Fracture

Fracture refers to a disruption in bone integrity, and its healing is an intricate regenerative cascade. Multiple factors influence fracture healing, including pathological factors such as aging and diabetes, as well as mechanical factors such as unstable fixation and insufficient mechanical loading 152. When the healing process is interrupted or delayed by these factors, it may result in delayed union and even progress to nonunion.

#### 4.2.1 Senescent cell in fracture

The most severe clinical consequence of osteoporosis is not bone loss itself, but a significantly elevated risk of fragility fractures 97, 153. For older adults, fractures often require a prolonged recovery period and carry an extremely high risk of various complications. Senescent cells are present not only in chronically degenerated bone tissue, but also increase rapidly during the healing phase after acute fracture 154. During the early stage of fracture healing, these cells are transiently present, and their early SASP factors help initiate the necessary inflammatory response and recruit reparative cells 41, 155. By contrast, persistent or excessive accumulation of senescent cells leads to sustained chronic inflammatory signaling. This disrupts the balance of the fracture repair microenvironment 41, weakens stem cell activity and osteogenic differentiation, and ultimately contributes to outcomes such as delayed union and non-union. Thus, appropriate coordination of the spatiotemporal dynamics of senescent cells may be an important determinant of successful fracture healing.

#### 4.2.2 Physiological characteristics of fracture healing

Fracture healing is a type of tissue regeneration highly similar to bone development and features scarless healing capacity 156. Despite the strong regenerative potential of bone tissue, this biological process may occasionally fail, leading to delayed fracture healing, pseudoarthrosis or non-union 157. Thus, further investigation into fracture healing has provided insights into novel targets that regulate the biologically optimized process of fracture repair, thereby offering valuable directions for future research aimed at preventing its failure. Given the investigation of senescent cells in the bone microenvironment, increased research has elucidated cellular and molecular alterations within this milieu, which subsequently impact bone remodeling as previously mentioned in the section of osteoporosis. Nevertheless, the impact exerted by these senescent cells and their function on the dynamic process of bone regeneration remains largely elusive.

The predominant mode of fracture healing in healthy individuals is indirect healing, which includes both endochondral and intramembranous bone formation 158. The process of indirect fracture healing encompasses four stages, namely hematoma formation, soft callus development, hard callus formation, and remodeling. In a fracture model of young adult mice, it has been observed that cellular senescence during bone healing exhibits a transient time course, reaching its peak approximately in the second week of fracture healing, which corresponds to the stage of soft callus formation in humans 159. Although senescent cells and their SASP exert beneficial physiological effects during skin wound healing in young individuals 66, compelling evidence demonstrating a positive role for SASP in bone healing remains lacking. This phenomenon may be attributed to the robust local inflammatory state following fracture masking the positive roles of senescent cells.

#### 4.2.3 Pathological mechanisms and intervention prospects of age-related impaired fracture healing

While increasing attention has been paid to the role of cellular senescence in aging, far less focus has been placed on age-related alterations in fracture healing. Indeed, the intricate nature of research design, encompassing comorbidities such as cardiovascular disease and diabetes mellitus, imposes a deleterious effect on bone healing during aging 160, 161. Thus, consistently identifying age as a potent risk factor for nonunion in clinical practice remains elusive 154. It is worth noting that the small animal model of fracture healing reveals a reduction in callus expansion, bone volume, and mechanical properties in aging mice 162, 163. Furthermore, by irradiating aged mice and transplanting young bone marrow into them, researchers observed that the older mice showed increased callus size and more bone during early healing stages, as well as faster callus remodeling in later stages of healing 164. Similar findings were observed in aged mice exposed to youthful circulation through heterochronic parabiosis 165. Therefore, investigation is warranted to elucidate the impact of cellular senescence on fracture healing during the aging process.

The hematoma formation stage during fracture healing is characterized by a robust early inflammatory response and hematoma formation at the fracture site. Senescent cells elicit SASP, which partially overlaps with the production of pro-inflammatory factors by inflammatory cells. Recently, it has been discovered that senescent cells in aged fracture healing not only exhibit the expression of SASP factors but also demonstrate upregulation of genes associated with ROS and DNA damage, thereby distinguishing them from inflammatory cells 166. Notably, the functional role of senescent cells in fracture repair is highly context- and age-dependent 159, 167. In young mice, senescent cells accumulate transiently during physiological fracture healing and peak at approximately 14 days post-fracture 159. Ablation of these short-lived senescent cells using senolytic agents such as D+Q markedly accelerates callus formation and enhances bone mechanical strength 159. In aged organisms, senescent cells accumulate persistently at the fracture callus and exert clear detrimental effects on the healing process 167. Insufficient mechanical loading, a common clinical issue leading to impaired healing, further disrupts this balance by downregulating Piezo1 expression in chondrocytes 168. Study has shown that such downregulation triggers aberrant accumulation of apolipoprotein E (ApoE), which in turn induces chondrocyte senescence, blocks chondrogenic-to-osteogenic transition, and ultimately delays fracture healing 168. This phenotype can be reversed by delivering Piezo1 agonists or local ApoE antagonists* via* injectable hydrogels. Furthermore, Senescent cells specifically localized within the fracture callus of aged mice secrete high levels of transforming growth factor-β1 (TGF-β1), a SASP factor that suppresses the proliferation and function of skeletal repair progenitor cells and serves as a key mediator of age-related fracture healing retardation 167. Neutralizing TGF-β1 or ablating senescent cells effectively alleviates this inhibitory microenvironment and restores bone regenerative capacity in aged individuals, whereas such interventions show no obvious beneficial effects in young counterparts 167. Further studies are needed to determine how the extent of senescent cell reduction influences bone repair in young individuals.

The role of inflammation modulation in aged fracture healing remains complex, particularly in relation to non-steroidal anti-inflammatory drugs (NSAIDs). Although suppression of age-related inflammatory signaling may reduce senescence and improve skeletal stem/progenitor cells (SSPCs) function, clinical evidence has also linked NSAID use after fragility fracture with an increased risk of subsequent fracture 169, 170. SSPCs can be considered as more homogeneous subsets of BMSCs, which function as osteochondro-precursors in callus formation during fracture healing 171, 172. The aging process is accompanied by a systemic and local proinflammatory environment, which contributes to age-associated cellular senescence as well as the decline in both number and function of SSPCs 169. Additionally, the senescent phenotypes exhibited by SSPCs themselves during the aging process constitute a significant contributing factor to the decline in bone regenerative potential associated with aging 173-175, which can be modulated through age-dependent secretion of grancalcin by macrophages during fracture healing 176. Therefore, the elimination of senescent SSPCs/BMSCs through approaches such as the utilization of senolytic drugs or gene knockout techniques may represent a promising strategy to enhance aged fracture healing 175, 177-179. However, further investigation is need to determine whether these treatments exert differential effects on fracture healing in aged individuals.

#### 4.2.4 Molecular heterogeneity of senescent cells

In recent years, a growing body of research has revealed that senescent cells exhibit marked molecular and functional heterogeneity during fracture healing. Specifically, as key senescence markers, p16^Ink4a^ and p21^Cip1^ transcripts exhibit marked heterogeneity across different cell types and tissues 180, 181. Moreover, existing study has identified tissue-specific differences in the SASP profiles associated with p21^Cip1^ or p16^Ink4a^ expression 180. Loss of p16^Ink4a^ comprehensively accelerates the healing process in aged individuals under chronic aging conditions, primarily by systemically activating the cyclin-dependent kinase 4/6 (CDK4/6)-pRB-E2F pathway, relieving the inhibition of BMSCs proliferation, and promoting their chondrogenic differentiation, osteogenic activation, and angiogenesis 182. In contrast, p21^Cip1^-expressing cells, which mainly comprise osteochondroprogenitors (OCHs) and specific neutrophil subpopulations, play a more direct and detrimental role in the skeletal injury response by secreting SASP factors 181. Functionally, eliminating p21^Cip1^-positive cells effectively improves fracture healing, whereas removing p16^Ink4a^-positive cells shows no direct effect on this process. By contrast, clearing p16^Ink4a^-positive cells alleviates age-related bone loss, while targeting p21^Cip1^-positive cells brings no beneficial results 181, 182. Collectively, these findings demonstrate that p16^Ink4a^-positive and p21^Cip1^-positive senescent cells differ in their occurrence contexts and pathological functions. Intervention strategies for fracture healing should take this heterogeneity into account **(Table 3)**.

### 4.3 ONFH

ONFH is a disease characterized by impaired blood supply, which leads to bone structural damage and femoral head collapse 184. ONFH can be classified into traumatic and non-traumatic forms. Traumatic ONFH is mainly caused by interruption of the blood supply after injury, which leads to cell death and tissue necrosis 185. Non-traumatic ONFH is more complex and is often associated with metabolic disorders, systemic diseases, excessive alcohol intake, and long-term corticosteroid use 186.

Synthetic steroid therapy can impair endothelial cell ribosomal RNA (rRNA) transcription and trigger cellular senescence in bone blood vessels 187. Recently, SA-β-gal activity was examined using X-gal staining on femoral head specimens from non-traumatic ONFH patients younger than 65 years, which helped minimize confounding effects caused by natural aging 188. The transitional zone between necrotic and healthy bone tissue in ONFH showed a distinct area with intense X-gal staining. Moreover, cellular senescence was observed in BMSCs, osteoblasts and osteocytes in these samples 188. A study has shown that both ONFH mouse models and glucocorticoid-treated BMSCs* in vitro* exhibit elevated senescence markers (e.g., SA-β-gal, p53, and p21^Cip1^) and reduced autophagy, as evidenced by decreased LC3 and increased p62 expression. These changes are accompanied by impaired osteogenic differentiation and enhanced adipogenic differentiation of BMSCs 189. Collectively, these findings suggest that glucocorticoid not only directly regulates BMSC fate but also indirectly influences their differentiation by inducing cellular senescence and disrupting autophagy, thereby contributing to the onset and progression of ONFH. Notably, metformin, a well-established anti-senescence agent 190, has been shown to downregulate senescence-associated genes in BMSCs and effectively inhibit ONFH progression* in vivo* 191. Therefore, the development of ONFH is closely associated with cellular senescence.

BMSCs possess self-renewal capacity and can differentiate into multiple cell lineages including osteoblasts and endothelial cells, thereby promoting bone regeneration and angiogenesis. Besides, BMSCs exert paracrine functions by secreting growth factors to enhance vascularization within necrotic regions 192. A series of clinical investigations have reported on the transplantation of BMSCs, which has progressively emerged as a promising therapeutic strategy for ONFH 193-195. However, transplanted BMSCs undergo substantial stress-induced apoptosis and senescence within the oxidative stress microenvironment associated with osteonecrosis of the femoral head, which in turn compromises their therapeutic efficacy 196, 197. In following study, it was discovered that the primary factor contributing to this microenvironment is the upregulation of p53, which effectively inhibits mitochondria translocation of Parkin 198. Thus, the upregulation of Parkin and downregulation of p53 were observed to enhance mitophagy in BMSCs, thereby facilitating the regenerative process in early steroid-induced ONFH. Additionally, the modulation of specific histone modifications can exert an influence on DNA methylation patterns and disrupt the nuclear homeostasis of MSCs, thereby also potentially attenuating senescence in BMSCs 199, 200. Further investigation is warranted to explore whether this modulation could enhance the efficacy of BMSC transplantation for early ONFH* in vivo*. Notably, although stem cell therapy for ONFH is associated with only mild complications 201, uncertainties still remain regarding its safety profile and the long-term behavior of transplanted cells. Conditioned medium derived from human mesenchymal stem cells offers improved safety and can exert anti-senescence effects by effectively inhibiting cellular senescent phenotypes 202, 203. Administration of MSC-CM prevents the progression of cellular senescence and SASP, while also effectively mitigating epiphyseal bone collapse in a murine model of ischemic osteonecrosis 188. These findings should be validated in a subsequent study using a large animal model of ONFH.

## 5. Cellular senescence in cartilaginous tissues

Cartilaginous tissues, such as articular cartilage and nucleus pulposus (NP) 204, play critical roles in the progression of pathologies including osteoarthritis (OA) and intervertebral disc degeneration (IDD). A unique feature of these tissues, compared with other tissues, is their avascular nature, which renders them incapable of effective intrinsic repair and poses substantial challenges to clinical repair and translational therapies 205. Consequently, dissecting the cellular and molecular events underlying their pathological progression is of particular importance. Similar to skeletal muscle and bone tissue, accumulating evidence indicates that senescent cells act as key drivers of progressive cartilaginous tissues degeneration.

### 5.1 Chondrocyte senescence in OA articular cartilage

OA is a degenerative disease that affects the whole joint. It involves synovial inflammation, subchondral bone remodeling, cartilage degeneration, and osteophyte formation 206, 207. OA is also a major cause of pain and disability worldwide, creating a large medical and socioeconomic burden 208, 209. Articular cartilage is an avascular and aneural connective tissue. Articular cartilage depends on chondrocytes and the ECM they produce. Chondrocytes are the only resident cells in cartilage, and they help maintain its structure and function 210. The ECM consists predominantly of type II collagen and proteoglycans, which endow the joint with load-bearing capacity and lubrication. However, an important cellular event during the onset and progression of OA is the accumulation of chondrocyte senescence 211. Chondrocyte senescence refers to an irreversible state of cell cycle arrest, which can be triggered by various stressors such as oxidative stress, mechanical overload and chronic inflammation 208, 212. This process is accompanied by SASP activation, marked by abundant secretion of pro-inflammatory cytokines and matrix-degrading enzymes. These factors disturb the homeostatic balance of the local joint microenvironment and accelerate the degradation of cartilage ECM 213. Therefore, in-depth exploration of the molecular mechanisms driving chondrocyte senescence is essential for elucidating the pathological characteristics of OA and developing innovative therapeutic strategies.

#### 5.1.1 Metabolic imbalance in chondrocyte senescence

Metabolic reprogramming is a characteristic feature of cellular senescence 208. In chondrocytes from osteoarthritic tissue, metabolic balance is disrupted, along with ROS accumulation and impaired mitochondrial and cellular metabolism. Senescent chondrocytes exhibit mitochondrial dysfunction, characterized by reduced mitochondrial membrane potential, impaired ATP production, and excessive ROS accumulation 214. These mitochondrial changes are linked to abnormalities in multiple upstream signaling pathways. For instance, PDZK1 deletion disrupts its binding with the mitochondrial enzyme 3-hydroxy-3-methylglutaryl-CoA synthase 2 (Hmgcs2), which in turn triggers mitochondrial dysfunction and promotes chondrocyte senescence under mechanical overload 215. In addition to disrupted intracellular signaling, extracellular mechanical changes also contribute to this pathological process. Increased ECM stiffness is a typical pathological feature of OA. The stiffened matrix suppresses histone deacetylase 3 (HDAC3) expression, which promotes Parkin acetylation and induces excessive mitophagy. This regulatory cascade ultimately accelerates chondrocyte senescence and exacerbates OA progression *in vivo* 216.

Downregulation of SIRT4 also markedly accelerates chondrocyte senescence and OA progression by impairing mitochondrial function 214. This occurs through suppressed Pink1 expression in chondrocytes, which impairs the removal of damaged mitochondria and aggravates intracellular metabolic dysfunction. As a central regulator of cellular metabolism and stress responses, SIRT1 also modulates chondrocyte senescence under inflammatory conditions. A study demonstrated that pro-inflammatory cytokines including tumor necrosis factor-α (TNF-α) and interleukin-1β (IL-1β) promote SIRT1 cleavage in chondrocytes. Restricted mitochondrial permeability limits cytochrome c release, increasing the intracellular ratio of 75-kDa SIRT1 to cytochrome c and allowing these senescent cells to maintain viability 217. Furthermore, the ratio of N-terminal to C-terminal SIRT1 in serum has been validated as a biomarker for early-stage OA and a predictor of therapeutic response to senolytics 217. Beyond SIRT4 and SIRT1, SIRT6, another sirtuin family member closely linked to metabolic homeostasis, also contributes essentially to chondrocyte senescence. Depletion of SIRT6 has been shown to exacerbate the senescent phenotype 218-220, potentially through mechanisms associated with dysregulated metabolic control.

Recent studies have shown that metabolic imbalance of trace elements including iron and selenium drives chondrocyte senescence, thereby exerting a critical impact on the initiation and progression of OA. Metabolic imbalance of iron, particularly iron overload, directly triggers a burst of intracellular ROS in chondrocytes, leading to oxidative stress, cell cycle arrest, and apoptosis, while suppressing matrix synthesis 221, 222. In contrast, treatment with the iron chelator deferoxamine (DFO) delays chondrocyte senescence and slows OA progression. DFO acts by suppressing NCOA4-mediated ferritinophagy and maintaining intracellular iron balance 223. Meanwhile, selenium exerts key antioxidant functions by incorporating into selenoproteins such as glutathione peroxidase 4 (GPX4), notably by eliminating lipid peroxides and protecting against ferroptosis 224. It has been reported that selenium phosphate synthetase 1 (SEPHS1) is downregulated in mouse and human OA cartilage, and that SEPHS1 downregulation weakens the ability of chondrocytes to synthesise selenoproteins with oxidoreductase function, thereby increasing ROS levels and promoting chondrocyte senescence 225.

Lipid metabolic imbalance is an important factor driving chondrocyte senescence, and its underlying mechanisms involve systemic dysregulation of fatty acid profiles and local abnormal lipid secretion. A study has shown that obesity-induced high-fat diets significantly increase the proportion of pro-inflammatory ω-6 fatty acids and decrease anti-inflammatory ω-3 fatty acids. This imbalanced ω-6/ω-3 ratio induces premature senescence of chondrocytes and exacerbates OA 226. In contrast, gene therapy-mediated delivery of the *fat-1* gene to correct this ratio effectively alleviates cellular senescence and joint degeneration.

#### 5.1.2 Changes of mechanical signalling pathways in chondrocyte senescence

Articular cartilage is exposed to mechanical loading throughout life, and appropriate mechanical stimulation is essential for maintaining cartilage homeostasis. However, abnormal mechanical stress is an important factor that predisposes to OA and can directly push chondrocytes into senescence.

Along with aging, mechanical overload is considered a major risk factor for OA 227. Persistent excessive loading on the joints speeds up cartilage degeneration and in turn contributes to OA progression 228. Accordingly, alterations in mechanical signaling pathways triggered by mechanical overload have attracted increasing attention in recent years. Studies across* in vitro* models, experimental OA mice, and human OA cartilage have collectively unraveled the mechanisms by which disrupted mechanical signalling promotes chondrocyte senescence and OA development.

Research has revealed that the role of mechanical stress in cartilage ageing and further explored its underlying mechanisms in OA development using* in vitro* systems, mouse models of experimental OA, and human OA cartilage specimens 229. They found that mechanical overload accelerated the senescence of chondrocytes, and that F-box and WD repeat domain containing 7 (FBXW7) exhibited reduced expression in chondrocytes under mechanical overload, which in turn induced upregulation of genes such as p16^Ink4a^ and p21^Cip1^, resulting in the exacerbation of OA 229. Additionally, Shao *et al*. found that mechanical overload reduces PDZ domain containing 1 (PDZK1) expression, and PDZK1 deficiency plays a pivotal role in mediating excessive mechanical overload-induced chondrocyte senescence in OA, with this effect closely associated with mitochondrial dysfunction 215. Meanwhile, Zhu *et al*. employed RNA sequencing analysis to compare chondrocytes treated with 20% elongation strain loading for 24 hours and untreated controls, identifying ribosomal protein L35 (RPL35) as another critical molecule; mechanical overload downregulates RPL35, and this downregulation promotes chondrocyte senescence and OA development, further expanding the repertoire of molecules involved in mechanical signalling pathway changes 230.

The transduction of mechanical stimuli into biochemical senescence signaling relies largely on mechanosensitive ion channels and interacts with multiple intrinsic regulatory cascades. As a typical mechanosensitive calcium channel, Piezo1 senses aberrant mechanical stress and triggers intracellular calcium overload, which further promotes the secretion of SASP components including IL-6 and IL-1β and amplifies the pro-senescent microenvironment within joint tissue 231. Beyond mechanical sensing channels, acid-sensing ion channel 1a (ASIC1a), a proton-activated cation channel abundantly expressed in chondrocytes, governs articular cavity pH homeostasis. Under acidic conditions at a pH of 6.0, ASIC1a mediates the downregulation of Lamin B1, modulates chondrocyte autophagosome, and consequently drives the progression of chondrocyte senescence 232.

It is worth noting that these mechanically activated pathways often intersect with key developmental signaling cascades. For instance, both the Hedgehog pathway, which can be activated after RPL35 downregulation, and the Notch pathway, whose abnormal activation is regulated by mechanical stress-associated proteins such as MYL3, are involved in stress-induced senescence 230, 233. This finding suggests that the pathogenesis of OA may partly result from inappropriate reactivation or dysregulation of developmental programs under abnormal mechanical conditions.

#### 5.1.3 Epigenetic modification related pathways in chondrocyte senescence

Epigenetic alterations contribute to both the stability and the plasticity of the senescent state and represent an important molecular basis for senescence heterogeneity 234. In osteoarthritic chondrocytes, epigenetic remodeling occurs in a dynamic and multilayered manner.

Among non-coding RNAs (ncRNAs), microRNAs (miRNAs) are major regulators in chondrocyte senescence and form a complex regulatory network. These endogenous single-stranded ncRNAs are typically 20-24 nucleotides long and mediate post-transcriptional regulation of gene expression 235. Increasing evidence indicates that numerous miRNAs are involved in cellular senescence and can either promote or inhibit chondrocyte senescence and OA development. Several studies have reported pro-senescent effects of specific miRNAs in chondrocytes. For instance, Liu *et al*. showed that miR-33-5p promotes OA-like cartilage degeneration by targeting SIRT6 mRNA for degradation, thereby accelerating chondrocyte senescence 236. Similarly, miR-99b-5p is significantly upregulated in OA cartilage and aggravates disease progression by directly inhibiting the expression of milk fat globule-epidermal growth factor 8 (MFG-E8). Since MFG-E8 can both delay chondrocyte senescence and drive macrophages toward an anti-inflammatory M2 phenotype, the miR-99b-5p/MFG-E8 axis may serve as a potential therapeutic target for OA 237.

In contrast, several miRNAs exhibit cartilage-protective effects. Zhu *et al*. demonstrated that miR-29b-5p shows markedly reduced expression in OA cartilage, whereas its restoration abrogates matrix metalloproteinases as well as senescence-associated genes including p16^Ink4a^ and p21^Cip1^* via* inhibiting ten-eleven-translocation enzyme 1 (TET1) 238. miR-26b-5p also confers protection against OA by attenuating the expression of asporin, which is elevated in OA chondrocytes and linked to chondrocyte senescence 239. Moreover, Si *et al*. found that miR-140 overexpression effectively reduced the levels of γH2AX, p53, p16^Ink4a^, p21^Cip1^, and SA-β-gal in OA chondrocytes, and delayed chondrocyte senescence and disease progression in OA rats 240. Notably, miR-653-5p is a chondroprotective miRNA that is significantly downregulated in osteoarthritic cartilage. Lin *et al*. identified miR-653-5p as a differentially expressed miRNA between OA and normal articular cartilage, demonstrating that its reduced expression in OA patient cartilage drives the enhanced senescent phenotype of articular chondrocytes and promotes OA pathogenesis 241.

Long ncRNAs (lncRNAs) and circular RNAs (circRNAs) represent crucial regulatory molecules governing chondrocyte senescence during OA progression. Certain lncRNAs promote senescence in joint tissues. Among them, ELDR accelerates chondrocyte senescence and promotes OA development. It forms a molecular complex with hnRNPL and KAT6A to modulate histone modification at the IHH gene promoter and further activate the Hedgehog signaling cascade 242. In contrast, specific lncRNAs exhibit protective properties against cartilage degeneration. The transcript lncRNA AC006064.4-201 functions as a novel biomarker and protective regulator. It interacts with PTBP1 to destabilize CDKN1B messenger RNA, thereby relieving chondrocyte senescence and mitigating OA injury 243.

Multiple circRNAs also participate in modulating this pathological process. Experimental evidence from* in vitro* and* in vivo* models confirms that circP4HA3 drives chondrocyte senescence through the miR-5001-5p/THBS2 signaling axis and aggravates OA deterioration 244. Moreover, elevated expression of circGNB1 has been detected in chondrocytes exposed to oxidative and inflammatory stress as well as in aged human cartilage tissues. Upregulated circGNB1 enhances the expression of catabolic factors, exacerbates intracellular oxidative stress, and suppresses anabolic gene profiles, ultimately promoting OA progression 245.

Compared with the above ncRNA subtypes, small-derived RNAs (sdRNAs) remain poorly investigated in the field. A representative molecule sdRNA-D43 originates from the small nucleolar RNA snoRD43. This sdRNA alleviates chondrocyte senescence and attenuates OA lesions. It targets both NRF1 and WIPI2 to negatively modulate the PINK1/Parkin-dependent mitophagy pathway 246. Collectively, these ncRNAs, including miRNAs, lncRNAs, circRNAs, and sdRNAs, exert either pro- or anti-senescent effects in chondrocytes and represent promising therapeutic targets for OA intervention **(Table 4)**.

Acetylation is a fundamental post-translational modification mediated by the transfer of acetyl groups from acetyl-CoA 250. The NAD+-dependent SIRT family of histone deacetylases is one of the major regulators of acetylation–deacetylation homeostasis 251. Clinically, the serum ratio of N-terminal to C-terminal SIRT1 peptides has been suggested as a potential indicator of early OA and chondrocyte senescence in both mouse and human samples 217. Moreover, dysregulated ubiquitination signaling is closely related to cartilage degeneration. TRIM15, an E3 ubiquitin ligase that is highly expressed in OA patients and aged mice, drives YAP nuclear translocation* via* K48-linked ubiquitination, which then activates the Hippo-YAP/TAZ pathway and promotes chondrocyte senescence and OA development 252. Notably, TRIM15 silencing by AAV5-delivered short hairpin RNA (shRNA) reduced OA progression in mice. In contrast, palmitoylation appears to exert a protective effect on chondrocyte homeostasis. The palmitoyltransferase ZDHHC11 facilitates the palmitoylation of apolipoprotein D (APOD), thereby protecting it from ubiquitin-mediated degradation in both DMM-induced and naturally aged mouse models. This mechanism contributes to the maintenance of chondrocyte metabolic homeostasis and mitigates OA-associated pathological changes 253. Emerging evidence has demonstrated that the methyltransferase-like 3 (METTL3) mediates m⁶A modification of ATG7 mRNA, a core autophagy-related gene, and promotes cellular senescence and OA progression* via* regulating the autophagy-GATA4 signaling axis 254. These findings illustrate that m⁶A methylation fine-tunes senescence-associated pathways at the post-transcriptional level, providing a novel epigenetic perspective for dissecting the molecular mechanisms underlying OA.

#### 5.1.4 Inflammatory signaling pathways in chondrocyte senescence

The inflammatory response is one of the important factors that promotes cellular senescence, and in turn, senescent cells secrete SASP components such as proinflammatory factors, which exacerbate joint inflammation 255. It has been reported that controlling senescence-related inflammation by targeting specific inflammatory mediators may have beneficial therapeutic effects in age-related diseases 256. Pro-inflammatory cytokines such as the IL-1β are closely associated with the onset of OA. A study demonstrated that IL-1β promotes the phosphorylation of TBK1 by activating the cGAS-STING and Toll-like receptor 3 (TLR3)-TRIF signaling pathways, which in turn activates the downstream NF-κB and MAPK pathways 257. This process promotes the expression of inflammatory mediators and catabolic enzymes such as MMP-13 and ADAMTS5, thereby triggering cellular senescence and inflammatory activation in chondrocytes while accelerating cartilage breakdown. In addition, Guo *et al*. demonstrated that STING expression was markedly increased in OA tissues and chondrocytes exposed to IL-1β, which enhanced the expression of senescence related genes in chondrocytes 258. Furthermore, in addition to IL-1β, TNF-α is another key regulatory factor of inflammation 259. A study has shown that gene transfer of TNF-α-induced protein 8-like protein 2 (TIPE2) can effectively delay the progression of OA in a premature aging *Zmpste24*^-/-^ mouse model by synergistically attenuating the inflammatory (TNF-α/NF-κB) pathway and the senescence (p16^Ink4a^/p21^Cip1^) pathway 260.

#### 5.1.5 Targeting chondrocyte senescence for OA treatment

##### 1) Gene therapy

To improve targeting specificity for senescent chondrocytes in OA, nucleic acid-based precision therapeutic strategies have been developed, with diverse approaches showing promising preclinical outcomes. Among these strategies, genetic tools based on the p16^Ink4a^ promoter are especially valuable because of their high specificity, as illustrated by two well-established transgenic mouse models. The INK-ATTAC model uses the p16^Ink4a^ promoter to drive expression of a modified FKBP-caspase-8 fusion protein, which can be dimerized and activated by the small molecule AP20187 to selectively induce apoptosis in senescent cells with high p16^Ink4a^ expression 261. Similarly, in p16-3MR transgenic mice, ganciclovir (GCV) can be used to selectively eliminate p16^Ink4a^-high senescent cells. Jeon *et al*. showed that AP20187-induced clearance of senescent cells reduced age-related OA in INK-ATTAC mice. In p16-3MR mice, local removal of senescent cells with GCV also reduced OA progression in post-traumatic joints. This treatment helped create a pro-regenerative microenvironment 262.

Senescent chondrocytes in OA have also been targeted using nucleic acid–based delivery methods. One example is AAV5-mediated delivery of shRNA against TRIM15. This approach reduced chondrocyte senescence and slowed disease progression. Similar effects were also observed in human cartilage explants. These findings suggest that targeting TRIM15 may be a promising strategy for OA treatment 252. Moreover, a study described a strategy that uses lipid nanoparticles (LNPs) to deliver ZDHHC11 mRNA, thereby enhancing protein palmitoylation and alleviating chondrocyte senescence 253. Stem cell-homing hydrogels have likewise been applied as a delivery system for miR-29b-5p, which can both suppress chondrocyte senescence and recruit endogenous stem cells to promote cartilage repair 238. Overall, these gene therapy strategies provide several ways to target senescent chondrocytes and slow OA progression.

##### 2) Senolytics

Senotherapy is being explored as a treatment for OA because it targets senescent cells. Among the available approaches, senolytic agents have received considerable attention for their ability to selectively remove these cells. The combination of D+Q was reported to reduce senescent cell burden and improve tissue function in naturally aged animals 263. In OA models, D+Q decreases the number of senescent chondrocytes and suppresses SASP production in vitro. Studies in knee OA and lumbar facet joint OA mouse models further showed that D+Q treatment reduces inflammation, promotes cartilage anabolism, and delays disease progression 264, 265. Notably, preliminary clinical data have also emerged supporting the potential of D+Q in human patients 266. Another representative senolytic, ABT-263, induces apoptosis in senescent chondrocytes by inhibiting B-cell lymphoma-2 (Bcl-2) family proteins, which in turn reduces inflammation and preserves the chondrocyte phenotype 267. Moreover, senescent cell clearance by ABT-263 has been shown to rescue the biological functions of synovial BMSCs in OA patients 268.

Despite these promising findings, current senolytic agents suffer from limited specificity. The anti-apoptotic pathways targeted by these compounds are also expressed in certain healthy cells, which can cause off-target adverse effects such as thrombocytopenia 269. Accordingly, the development of targeted delivery systems relying on senescent cell-specific surface antigens, as well as prodrug strategies that take advantage of their heightened lysosomal activity, has become a critical direction to widen the therapeutic window of senolytic interventions for OA.

##### 3) Senomorphics

In addition to senolytic clearance of senescent chondrocytes, senomorphic therapy, which suppresses the harmful SASP of senescent cells without eliminating them, represents another critical therapeutic strategy for OA 20, 208. This approach targets either the upstream signaling cascades that drive SASP production in senescent chondrocytes, including NF-κB, p38 MAP kinase, and mTOR, or the downstream effector molecules that directly mediate cartilage damage and inflammation in OA joints 208.

Among upstream pathway inhibitors, the mTOR inhibitor RAPA stands as a classic senomorphic agent, which suppresses SASP production without eliminating senescent cells 270, 271. In a preclinical study, intra-articular administration of RAPA-loaded PLGA microparticles was shown to effectively induce autophagy, lessen chondrocyte senescence, and decrease proinflammatory cytokine secretion, thereby reducing cartilage damage in murine OA models 272. Mechanistically, RAPA promotes transcription factor EB (TFEB) nuclear translocation and augments autophagic flux* via* mTOR pathway inhibition, further blunting SASP release to slow OA progression 270. Besides RAPA, metformin, a commonly used first-line oral antidiabetic drug, also has marked senomorphic effects in OA chondrocytes. It is generally considered to act through AMPK activation, which suppresses mTOR signaling and reduces oxidative stress, thus improving key aging-related changes 190. Yan *et al*. found that metformin regulates miRNA-34a expression and upregulates its target gene SIRT1, thereby reducing senescence, promoting proliferation, and restoring ECM homeostasis in OA chondrocytes 273.

**Figure 4:**
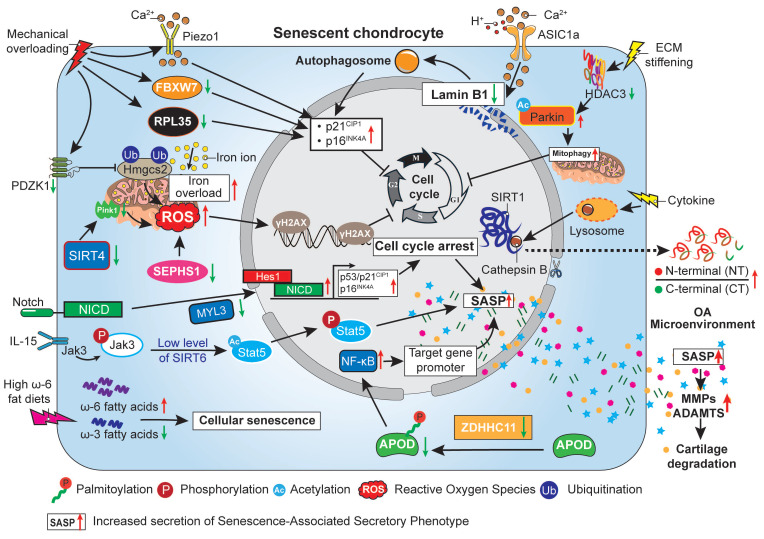
** The molecular mechanisms of senescent chondrocyte in the osteoarthritis (OA) microenvironment.** Mechanical overload induces chondrocyte senescence and OA progression through multiple mechanisms: mechanical overload reduces PDZ domain containing 1 (PDZK1) expression, which impairs chondrocyte mitochondrial function and triggers ROS accumulation by protecting 3-hydroxy-3-methylglutaryl-CoA synthase 2 (HMGCS2) from ubiquitination; reduces F-box and WD repeat domain containing 7 (FBXW7) to upregulate p16^Ink4a^ and p21^Cip1^; and decreases ribosomal protein L35 (RPL35) to promote senescence. In addition, the mechanosensitive channel PIEZO1 mediates Ca^2+^ influx in response to mechanical stress and promotes chondrocyte senescence. Under acidic conditions, acid-sensing ion channel 1a (ASIC1a) mediates the downregulation of lamin B1, modulates chondrocyte autophagosome, and consequently drives the progression of chondrocyte senescence. Stiffened extracellular matrix extracellular matrix (ECM) stiffening downregulates the expression of histone deacetylase 3 (HDAC3), promotes the acetylation of Parkin, thereby inducing excessive mitophagy and accelerating senescence in mouse chondrocytes. Metabolic iron imbalance, especially iron overload, directly induces a burst of intracellular ROS in chondrocytes, leading to cell cycle arrest. Downregulation of sirtuin4 (Sirt4) in chondrocytes inhibits PINK1 activity, impairs the cellular capacity to clear damaged mitochondria, thereby exacerbating ROS accumulation and accelerating chondrocyte senescence. Selenium phosphate synthetase 1 (SEPHS1) downregulation weakens the ability of chondrocytes to synthesise selenoproteins with oxidoreductase function, thereby increasing ROS levels and promoting chondrocyte senescence. The reduction of Myosin light chain 3 (MYL3) induces Notch receptor internalization and Notch intracellular domain (NICD) nuclear translocation, ultimately activating the Notch signaling pathway and triggering chondrocyte senescence. Low level of Sirt6 regulates senescence by modulating signal transducer and activation of transcription 5 (STAT5) phosphorylation, nuclear translocation, and transcriptional activity, which ultimately activates the IL-15/JAK3/STAT5 signaling pathway and exacerbates chondrocyte senescence. Sirtuin-1 (SIRT1) is cleaved by cathepsin B into an N-terminal (NT) polypeptide (75SIRT1) and a C-terminal (CT) fragment, and an increased NT/CT ratio reflects chondrocyte senescence. ZDHHC11 deficiency decreases apolipoprotein D (APOD) palmitoylation, resulting in NF-κB upregulation at target gene promoters and ultimately leading to chondrocyte senescence. A diet rich in ω-6 fatty acids elevates the cellular ratio of ω-6 to ω-3 fatty acids, thereby exacerbating chondrocyte senescence. Senescent chondrocytes contribute to the OA microenvironment by secreting SASP factors. Among these, elevated matrix metalloproteinases (MMPs) and a disintegrin and metalloproteinase with thrombospondin motifs (ADAMTS) drive cartilage degradation. Red upward arrows indicate upregulation, and green downward arrows indicate downregulation.

Targeting downstream effector molecules of the SASP represents an alternative senomodulatory approach. This therapeutic strategy is designed to suppress pivotal mediators responsible for cartilage destruction in OA, encompassing pro-inflammatory chemokines, growth factors, cytokines, as well as matrix-degrading proteases 274. Nevertheless, early clinical investigations focusing on single SASP components fail to yield satisfactory clinical outcomes for arresting OA progression. Notably, Faust *et al*. showed that intra-articular injection of anti-interleukin-17 (IL-17) neutralizing antibodies effectively alleviates joint degenerative lesions and eliminates accumulated senescent cells in murine models of post-traumatic OA. To date, the clinical efficacy of the IL-17-targeted monoclonal antibody secukinumab in patients with knee OA remains under evaluation 275. Collectively, senomorphic strategies targeting SASP in senescent chondrocytes provide a valuable complementary approach to senolytic clearance for OA treatment** (Figure 4)**.

### 5.2 IDD

Low back pain is a leading musculoskeletal disorder worldwide, with around 40% of cases caused by IDD and lumbar disc herniation 276. The intervertebral disc is an avascular fibrocartilaginous tissue between adjacent vertebrae, composed of the nucleus pulposus (NP), annulus fibrosus (AF), and cartilaginous endplate (CEP), and functions in shock absorption, spinal motion preservation, and distribution of axial and torsional forces. Its avascularity, low cellularity, mechanical stress, and limited nutrient transport render it particularly susceptible to injury 277. The pathological features of IDD include gradual dehydration of NP, loss of AF layered structure and mechanical integrity, and a reduction in normal elasticity and tensile strength of IDD 278.

#### 5.2.1 Triggers of NPC senescence in IDD

**Figure 5:**
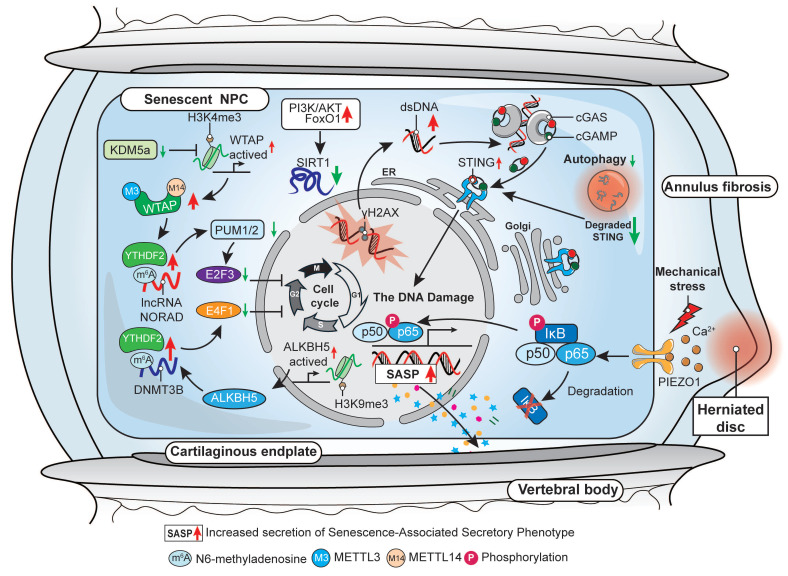
** Molecular regulatory mechanisms underlying nucleus pulposus cell (NPC) senescence.** In senescent NPCs, lower KDM5a expression enhances H3K4me3 modification at the WT1 associated protein (WTAP) promoter, resulting in increased WTAP expression. The increase in WTAP further promotes m^6^A methylation of NORAD RNA, which is then recognized and degraded by YTHDF2. The decline in NORAD transcripts reduces the sequestration of the RNA-binding proteins Pumilio 1/2 (PUM1/2), allowing more PUM1/2 to bind E2F3 mRNA. This interaction promotes the recognition and degradation of E2F3 transcripts and accelerates NPC senescence. ALKBH5 also promotes NPC senescence by demethylating DNMT3B mRNA. Reduced m6A methylation impairs YTHDF2-mediated degradation of DNMT3B transcripts and increases DNMT3B expression. DNMT3B then methylates the E4F1 promoter, which suppresses E4F1 transcription and protein expression. In NPCs, mechanical stress triggers NF-κB p65 activation through the PIEZO1-Ca^2+^ axis, thereby elevating periostin at the transcriptional level. Notably, periostin released by senescent NPCs can further enhance NF-κB p65 activity, thereby establishing a persistent pro-senescent feedback loop. Impaired autophagic degradation of STING results in its abnormal accumulation, creating a vicious cycle that promotes cellular senescence. In addition, aberrant genomic DNA damage activates the cGAS–STING axis, driving inflammatory senescence in NPCs. Moreover, the protective effect of SIRT1 against oxidative stress-induced senescence in NPC is regulated by the Akt-FoxO1 pathway. Red upward arrows indicate upregulation, and green downward arrows indicate downregulation. ER: endoplasmic reticulum; Golgi: Golgi apparatus.

Nucleus pulposus cells (NPCs) are the primary functional cells within the intervertebral disc, responsible for synthesizing and maintaining an ECM rich in proteoglycans and type II collagen, thereby preserving disc elasticity and load-bearing capacity. Numerous studies have shown that senescent NPCs are a key pathogenic contributor to IDD **(Figure 5)**.

Multiple stressors in the disc microenvironment can directly induce or accelerate NPC senescence, a key initiating event in IDD. Among these, excessive accumulation of ROS is a major trigger of NPC senescence in IDD. Under ROS stress, the PI3K/Akt-FoxO1 pathway is activated, which in turn suppresses SIRT1 expression and induces NPC senescence 279. Functional materials targeting ROS clearance have demonstrated therapeutic potential, and glutathione-doped carbon dot nanozymes can scavenge excessive ROS, alleviate oxidative stress-induced mitochondrial damage, inhibit NPC senescence and mitigate the progression of IDD 280. Similarly, loss of glutathione peroxidase 3 (GLRX3) in NPCs leads to ROS accumulation, whereas delivery of MSC-derived EVs enriched with GLRX3 (EVs-GLRX3) effectively clears ROS, preserves mitochondrial function, and suppresses NPC senescence 281. Moreover, exosomes from polarized M1 macrophages induce NPC senescence, activating the NF-κB pathway and upregulating SASP factors and matrix-degrading enzymes, which disrupt ECM homeostasis and accelerate IDD 282. The abnormal accumulation of metabolites in degenerated discs promotes NPC senescence, with lactate playing a central role. Lactate interacts with the PH domain of Akt, inhibiting its phosphorylation and suppressing the PI3K/Akt pathway. Consequently, the Akt/p21CIP1/p27/cyclin D1 and Akt/Nrf2/HO-1 pathways are disrupted, leading to mitochondrial damage, ROS accumulation, and NPC senescence 283.

The specific nutritional-deficient microenvironment of the intervertebral disc is another key factor promoting NPC senescence. Kouroumalis *et al*. reported that when young NPCs were cultured under conditions mimicking the IDD microenvironment, including low glucose, hypoxia, high osmolality, and serum deprivation, their proliferative capacity was significantly inhibited. Meanwhile, senescence markers such as p16^INK4a^ and p21^CIP1^, as well as SASP-associated ECM-degrading enzymes were stably upregulated in senescent NPCs 284. Besides nutrient deficiency, unphysiological mechanical stimulation also promotes NPC senescence through activation of specific signaling pathways. Unbiased mRNA sequencing and assay for transposase-accessible chromatin sequencing (ATAC-seq) studies showed that mechanical stress initiates a self-amplifying loop of NF-κB and periostin through the mechanosensor PIEZO1, which in turn accelerates mechano-induced NPC senescence and the progression of IDD 285. In addition, prolonged exposure to cyclic mechanical tension (CMT) with unphysiological magnitude can activate the p53/p21^CIP1^-Rb pathway, not only inducing premature senescence of NPCs but also disrupting the structural and functional balance of the intervertebral disc 286.

#### 5.2.2 Regulatory mechanisms of NPC senescence

NPC senescence in IDD is governed by a complex network of molecular mechanisms. Emerging evidence indicates that ncRNAs, particularly lncRNAs, modulate NPC senescence through competing endogenous RNA (ceRNA) crosstalk or trans-regulatory mechanisms, analogous to their roles in OA-related chondrocyte senescence. For example, HOTAIR, a lncRNA, is positively correlated with the severity of IDD. Ectopic overexpression of HOTAIR enhances autophagy in NPCs, thereby accelerating apoptosis, cellular senescence, and ECM catabolism. These findings suggest that HOTAIR silencing may represent a promising therapeutic strategy for IDD 287. Another lncRNA, TRPC7-AS1, is upregulated in IDD and functions as a ceRNA to sponge miR-4769-5p, thereby abrogating the inhibitory effect of miR-4769-5p on heparanase. The resulting upregulation of heparanase further accelerates NPC senescence, reduces cell viability, and disturbs ECM homeostasis 288. Moreover, in human samples and mouse models of IDD, studies have found that increased m^6^A methylation modification of lncRNA NORAD mediated by WT1 associated protein (WTAP) is a key factor leading to the senescence of NPCs. These senescent cells impede cell cycle progression by reducing the sequestration of PUMILIO proteins, which in turn enhances the degradation of E2F3 mRNA. Interventions targeting NORAD m6A modification or the NORAD/PUMILIO/E2F3 axis may thus represent potential strategies to alleviate IDD by preventing NPC senescence 289. lnc-HRK-2:1 upregulation drives IDD by trans-regulating downstream targets, identifying it as a potential therapeutic target 290. Under oxidative stress, the lncRNA H19 functions as a ceRNA to sequester miR-22, thereby attenuating its suppressive effect on lymphoid enhancer factor 1 (LEF1). This cascade leads to excessive activation of the Wnt/β-catenin pathway, ultimately driving senescence in NPCs and destabilizing the ECM 291.

Autophagy is a conserved intracellular degradation process that removes damaged organelles and misfolded proteins through lysosomal degradation, thereby preventing intracellular waste accumulation 83. Impairment of autophagy, including selective autophagy, chaperone-mediated autophagy (CMA), and mitophagy, accelerates NPC senescence. Enhancing autophagic activity may therefore provide therapeutic benefits. CMA is especially important because it protects NPCs from premature senescence by selectively degrading specific proteins. Using a rat caudal IDD model induced by intradiscal TNF injection, Cheng *et al*. demonstrated that reduced expression of LAMP2A, a critical regulator of CMA, compromises the degradation of phospholipase Cγ1 (PLCG1). This leads to PLCG1 accumulation, increased IP3 production, calcium release from the endoplasmic reticulum, and calcium overload, which ultimately induces NPC senescence 292. Consistent with these findings, a subsequent study demonstrated that CMA dysfunction contributes to a self-perpetuating degenerative cycle within intervertebral discs. Senescent cells exhibit DYRK1A accumulation accompanied by enhanced SASP production. In contrast, CMA activation counteracts these changes by promoting DYRK1A degradation, maintaining cellular homeostasis, and facilitating the selective elimination of senescent cells, thereby mitigating IDD progression 293. Besides CMA, selective autophagy receptors also affect NPC fate. For example, lower expression of the selective autophagy receptor NBR1 induces NPC senescence in both stress-induced human NPC models and rodent IDD models. These senescent cells exacerbate IDD by secreting SASP and disrupting ECM homeostasis, whereas NBR1 overexpression enhances clearance of the harmful protein SRBD1, delaying senescence and alleviating IDD 294. UCHL1, a deubiquitinating enzyme, serves as a key molecular mediator connecting autophagy and ferroptosis. Loss of UCHL1 expression induces HPCAL1-dependent autophagic ferroptosis, resulting in impaired cellular homeostasis. Conversely, restoration of UCHL1 through engineered exosome-based plasmid delivery reactivates chaperone-mediated autophagy, inhibits ferroptotic cell death, and promotes the clearance of senescent NPCs, ultimately alleviating IDD progression 295. Epigenetic mechanisms also interact closely with autophagy in the regulation of NPC senescence. Increased expression of the epigenetic regulator KMT2A accelerates NPC senescence by inhibiting autophagic activity, impairing ATG4a-dependent autophagic flux, and promoting GATA4 signaling. Conversely, depletion of KMT2A or METTL3 counteracts these alterations, alleviates the detrimental effects of senescent cells, and slows the progression of IDD 296. Mitophagy, the selective removal of damaged mitochondria, also contributes to this process. EGR1 knockdown by sh-EGR1 activates the PINK1-Parkin pathway, increases mitophagy, facilitates the clearance of damaged mitochondria, and inhibits NPC senescence 297. Collectively, modulation of these autophagic pathways may represent a promising approach for alleviating IDD by limiting NPC senescence.

#### 5.2.3 Therapeutic strategies targeting NPC senescence in IDD

Drugs targeting senescent NPCs may help treat IDD by reducing senescence and SASP production. Several senolytic compounds, particularly quercetin, have shown therapeutic potential in IDD. Quercetin activates Nrf2 signaling and inhibits NF-κB signaling, thereby reducing NPC senescence and slowing the progression of IDD 298. Resveratrol inhibits Akt phosphorylation activity, alleviating Akt-mediated suppression of FoxO1 to promote FoxO1 nuclear localization and transcriptional activity, ultimately upregulating SIRT1 expression to counteract oxidative stress-induced NPC senescence in rats 279. Dehydrocostus lactone (DHE), a natural compound, reduces NPC senescence and inflammation by inhibiting the STING-TBK1/NF-κB and MAPK pathways, and it significantly improves IDD in both *in vitro* and *in vivo* models 299. Curcumin and its metabolite o-Vanillin likewise exhibit selective senolytic activity against senescent human disc cells *in vitro*. They promote apoptosis of senescent cells, decrease SASP release, and enhance the proliferation and ECM synthesis of the remaining healthy cells by regulating the Nrf2 and NF-κB pathways 300. In addition, o-Vanillin has been shown to attenuate TLR-2-induced senescence in human NPCs, accompanied by reduced SASP production and enhanced matrix synthesis. These findings support its potential as a disease-modifying therapeutic agent for IDD and low back pain 301.

In addition to monotherapy, combination strategies targeting multiple senescence-related pathways have also been explored. Long-term intermittent treatment with D+Q reduces senescent cell accumulation and alleviates age-related IDD in mice. The therapeutic effect is strongly influenced by treatment timing, with earlier intervention showing better outcomes than treatment at advanced stages of degeneration 302. Combined treatment with RG-7112 and o-Vanillin shows stronger effects than monotherapy. It reduces senescent cell accumulation, inflammatory cytokine production, and neuronal axon outgrowth, suggesting potential benefits for discogenic low back pain 303. Localized delivery systems improve drug retention within the disc. In rat and goat IDD models, an anti-swelling hydrogel loaded with NPC exosomes and D+Q cleared senescent NPCs, reduced inflammation, and alleviated IDD 304. One recently developed strategy uses Cavin2-modified exosomes carrying the pluripotency factors Oct4, Klf4, and Sox2. This delivery system lowers senescence marker levels in NPCs, reduces DNA damage, restores proliferation and metabolic balance, and markedly preserves disc structure and function in animal models, suggesting a new way to rejuvenate senescent NPCs beyond conventional senolytic clearance 305.

Among exosome-based therapies, Liu *et al*. found that GLRX3-enriched exosomes derived from MSCs effectively clear ROS in NPCs, maintain mitochondrial function, and inhibit cellular senescence. By combining antioxidant effects with targeted delivery, this approach helps alleviate IDD in preclinical models 281. In another study using a rat IDD model, Sun *et al*. injected small EVs from induced pluripotent stem cell-derived MSCs into punctured intervertebral discs. These EVs delivered exogenous miR-105-5p, rejuvenated senescent NPCs, and reduced IDD progression 306. Moreover, LCN2-enriched exosomes released from M1 macrophages have been shown to activate NF-κB signaling, thereby promoting NPC senescence and aggravating IDD. Rather than serving as a therapeutic option, these results point to key pro-degenerative factors that should be taken into account and avoided in the development of biological therapies 282. At the same time, combining stem cell plasticity with traditional Chinese medicine has shown therapeutic potential. Bushen Huoxue Formula-containing serum, when used together with adipose-derived MSCs, activates the TGF-β1/Smad pathway, drives the differentiation of adipose-derived MSCs into NP-like cells, and delays oxidative stress-induced senescence in NPCs. In this way, it helps repair damaged NPCs and improve the local microenvironment, suggesting a new integrative approach for IDD 307.

Moreover, targeting the core drivers of NPC senescence may produce precise therapeutic benefits. Studies using 22-month-old wild-type C57BL/6 mice and p16-3MR transgenic mice found that systemic removal of p16^INK4a^-positive senescent cells can effectively alleviate age-related IDD, indicating that cellular senescence plays a causal role in IDD progression 308. Meanwhile, deficiency of p16^INK4a^ reduces oxidative stress in NPCs and promotes cell cycle progression, which further slows degeneration. Together, these results indicate that p16^INK4a^ functions not only as a biomarker, but also as an important driver of IDD, supporting gene-targeted modulation of p16^INK4a^ as a potential therapeutic approach 309.

#### 5.2.4 Cellular senescence of CEP in IDD

The CEP is a layer of hyaline cartilage located between the NP and the vertebral body, consisting primarily of chondrocytes embedded within a cartilage ECM. It plays an important role in distributing mechanical loads and protecting the vertebral bodies from stress during spinal motion. However, sustained mechanical loading can lead to CEP injury and degeneration, thereby contributing to the progression of IDD 310.

Disturbances in lipid metabolism have been identified in both high-fat diet-induced rat models of IDD and degenerative human CEP tissues, accompanied by elevated levels of oxidized low-density lipoprotein (ox-LDL). Through binding to the LOX-1 receptor, ox-LDL triggers activation of the ROS/p38-MAPK/NF-κB signaling cascade, thereby driving CEP chondrocyte senescence and calcification. Knockdown or pharmacological inhibition of LOX-1 can block this pathway, reduce senescence and calcification, and thereby alleviate disc degeneration 311. Beyond metabolic disturbances, mechanical stress is also implicated in the senescence of CEP chondrocytes. Intermittent cyclic mechanical tension (ICMT) suppresses YAP1 expression, promoting cellular senescence and degenerative changes in CEP chondrocytes. These alterations contribute to ECM degradation and accelerate intervertebral disc degeneration. Conversely, AAV-mediated YAP1 overexpression alleviates senescence-associated phenotypes, preserves cellular function, and mitigates ICMT-induced senescence and disc degeneration 312. Beyond its direct effects on chondrocytes, senescent osteoclasts in these models also produce excessive neurotrophic factors, including Netrin-1 and NGF, which promote abnormal sensory nerve ingrowth into porous endplates and contribute to spinal pain. Importantly, the senolytic agent ABT-263 can clear senescent osteoclasts, reduce CEP porosity, sensory innervation, and type H vessel growth, and thereby alleviate spinal hypersensitivity and disc degeneration 313.

#### 5.2.5 AF cell senescence in IDD

The AF is composed of concentric fibrocartilaginous layers that surround the NP and maintain disc integrity. AF cell senescence plays an important role in IDD progression. Mechanical stress, environmental factors, and inflammatory mediators can induce this process by activating senescence-related signaling pathways and promoting disc degeneration.

Mechanical overload is an important contributor to AF cell senescence. In a rat AF cell stretch model, high-magnitude mechanical strain activated the RhoA/ROCK pathway, resulting in cellular senescence and reduced proliferation. The ROCK inhibitor RKI-1447 partially alleviated these senescent phenotypes, indicating that RhoA/ROCK inhibition may be a potential strategy for relieving mechanically induced IDD 314. Oxidative stress and proinflammatory factors are also important in promoting AF cell senescence. In both rat IDD models and cultured AF cells, advanced oxidation protein products (AOPPs) have been reported to induce senescence* via* a NOX4-dependent MAPK signaling pathway. In this context, senescent cells display higher levels of p53/p21^CIP1^, p16^INK4a^, and proinflammatory cytokines, which further drive ECM breakdown. Using NOX4 inhibitors or specific shRNA to block this pathway alleviated senescence and inflammation and slowed IDD progression 315. Similarly, in rat AF cells, a TNF-α-induced inflammatory microenvironment activated the ROS/NF-κB pathway and promoted senescence. Treatment with melatonin inhibited ROS/NF-κB signaling, partly reversed inflammation-induced senescence, and suggested potential therapeutic value for inflammatory IDD 316. Environmental and physical stressors also contribute to AF cell senescence through distinct signaling pathways. In cultured AF cells, cadmium activates the JNK/p53 pathway and induces cellular senescence together with mitochondrial dysfunction. The associated SASP then further promotes ECM degradation. Blocking JNK suppresses this pathway and reduces senescence, indicating that it may be a useful target in cadmium-induced IDD 317. In rat and human AF cell cultures and in mouse models *in vivo*, ionizing radiation has also been shown to induce AF cell senescence through MMP-dependent signaling, along with increased matrix catabolism. These results suggest that limiting avoidable radiation exposure may help prevent radiation-related IDD 318.

Taken together, these studies show that AF cell senescence in IDD is controlled by multiple pathways that respond to mechanical, oxidative, inflammatory, and environmental stress. Targeting these context-dependent signaling pathways may help reduce AF cell senescence and relieve IDD.

## 6. Conclusion and Perspective

In this review, we present a systematic overview of the diverse roles of senescent cells and their impact on tissue repair involving three tissue types and seven prevalent musculoskeletal diseases. We describe the heterogeneous features of cellular senescence in different musculoskeletal tissues, stress the dual nature of senescent cells, summarize current therapeutic strategies targeting senescence, and outline the main barriers to clinical translation.

Cellular senescence is a dynamic process that involves broad changes in cellular physiology and molecular networks, eventually leading to alterations in cell morphology, structure, and function. Accumulating evidence has shown that senescent cells exhibit marked heterogeneity across distinct tissues, pathological conditions, and even within the same tissue microenvironment 37. This heterogeneity arises from the combined action of multiple factors, primarily senescence-inducing stress types, cellular origin and identity, specific transcriptomic and metabolic status of cells, as well as signals derived from the surrounding microenvironment 319. Cellular senescence should not be evaluated using only single markers or isolated cell populations. Bulk and single-cell transcriptomic analyses can better reveal cellular heterogeneity and senescence-related changes in cell communication and the microenvironment 8, 37. A study showed that senescent cells include different subpopulations. The six senescence identities (SIDs) defined by the SenCID framework have distinct transcriptional features, functions, and therapeutic responses 8. This heterogeneity also provides opportunities to develop therapies targeting specific senescent cell subpopulations.

Age-related disorders frequently coexist and are linked by overlapping cellular and molecular mechanisms. Among these, ROS has emerged as a key upstream factor capable of inducing senescence across multiple cell types. In bone tissue, excessive ROS accumulation directly causes mitochondrial dysfunction and oxidative stress in osteoblasts and BMSCs, which in turn leads to cellular senescence and reduced osteogenic capacity 115. Similarly, in skeletal muscle, increased ROS production triggered by factors such as ET-1 or acute injury accelerates FAP senescence and impairs their regenerative functions 52, 84. Collectively, these observations indicate that redox imbalance acts as a major inducer of cellular senescence across multiple tissues. Furthermore, the accumulation of senescent cells contributes largely to tissue dysfunction through the SASP, fostering a microenvironment that favors degeneration. In osteoporosis, SASP factors secreted by senescent osteoblasts, osteocytes, or BMSCs disrupt bone homeostasis by inhibiting osteoblast differentiation, promoting osteoclastogenesis, and skewing BMSC fate toward adipogenesis 28, 135. A comparable phenomenon has been reported in sarcopenia. Senescent FAPs and muscle stem cells produce a SASP rich in pro-inflammatory cytokines and profibrotic mediators, including CCL2 and OPN. These factors impair myoblast differentiation, promote fibrotic remodeling, and alter immune cell recruitment, thereby compromising muscle regeneration and functional maintenance. 74, 80. In addition, this crosstalk may also occur at the systemic level. For example, EVs derived from senescent BMSCs can be taken up by MuSCs and further worsen sarcopenia 320. These findings suggest that SASP acts not only as a local disruptor, but also as a possible mediator of inter-tissue communication in musculoskeletal aging.

As understanding of cellular senescence continues to grow, clinical strategies targeting senescent cells have become an important focus in translational medicine 4, 321. Although preclinical studies have shown that the removal of senescent cells can mitigate a range of age-related disorders, substantial obstacles remain before these findings can be translated into clinical practice. One major challenge is the pronounced heterogeneity of senescent cells. Their molecular characteristics and survival mechanisms differ according to cell type, tissue context, and the nature of the senescence-inducing stress, making it difficult for any single therapeutic strategy to effectively target all senescent cell populations 1. Second, some adverse effects of current senolytic agents have been reported in clinical trials, including thrombocytopenia, neutropenia, and cytokine release syndrome 15, 142, 322. Further optimization of drug selectivity is still required. Dosing regimens also need improvement to achieve a better balance between efficacy and safety.

Targeting senescent cells within the musculoskeletal system still presents several major challenges. First, senescent cells in this system are highly heterogeneous, with different senotypes displaying distinct molecular markers. This makes it difficult to accurately characterize, specifically target or eliminate these cells using only classic markers such as p16^Ink4a^ or p21^Cip1^. A promising strategy to address this barrier is to develop targeted interventions based on the unique microenvironmental dependencies of senescent cells. A study has shown that hypobaric pressure (-375 mmHg), without accompanying hypoxia, can induce lysosome-dependent cell death (LDCD) and selectively clear senescent cells. In addition, intermittent hypobaric pressure treatment prolonged lifespan and relieved osteoporosis in elderly mice, supporting hypobaric pressure as a natural senolytic strategy 323. Second, senescence in the musculoskeletal system is not invariably detrimental. In particular, during the early phase of tissue repair, transient and pro-remodeling senescence-like cells may emerge and contribute to regeneration. Accordingly, during the acute injury or early repair stage, priority should be given to tissue-specific senomorphic or SASP-modulating approaches in order to preserve the beneficial, short-term effects of senescence 324. In contrast, during chronic degeneration or the later phase of repair, intermittent senolysis may be applied to eliminate persistently deleterious senescent cell populations, which may also help reduce the side effects of senotherapeutic drugs 325. Finally, many classical senolytics were not specifically designed for the musculoskeletal system, and systemic senolytic therapy therefore faces challenges related to toxicity, off-target effects, and uncertain clinical translation. For example, although UBX0101, a p53/MDM2 inhibitor, showed beneficial effects in aged mice with post-traumatic OA 326, its phase II trial in patients reduced pain without significantly slowing disease progression 327. To overcome the limitations of systemic senolytic therapy, responsive local delivery systems have been developed. For example, the supramolecular nanoplatform Asp_10_SAC4A enables fracture-targeted and hypoxia-triggered release of D+Q, thereby promoting selective early clearance of senescent cells 328. In addition, identifying disease-specific deleterious senescent cell markers in the musculoskeletal system and developing targeted interventions against them represents another promising strategy. Recently, the urokinase-type plasminogen activator receptor (uPAR) has emerged as a relatively selective biomarker that distinguishes senescent cells from healthy cells in specific disease settings. Furthermore, uPAR-targeted CAR-T cells exhibit potent therapeutic efficacy in ablating senescent cells within mouse models of age-related diseases, including cancer and liver fibrosis, underscoring the therapeutic potential of disease-specific, marker-guided senescent cell targeting 322, 329. Notably, uPAR expression has been implicated in collagen-induced arthritis 330, and antibody-based inhibition of the proteolytic activity of urokinase-type plasminogen activator (uPA) can alleviate disease progression in mouse models of arthritis 331. Nevertheless, the presence, phenotypic features, and functional significance of uPAR-positive cells within the framework of cellular senescence in the musculoskeletal system have yet to be clarified.

Precision drug delivery is one of the key strategies to enhance the targeting and safety of anti-senescence therapies. Taking nanocarrier systems as an example, efficient encapsulation and targeted delivery of small-molecule senolytic drugs such as fenofibrate or nucleic acid-based drugs can be achieved by designing liposomes, polymeric nanoparticles, exosomes or other carriers with specific physicochemical properties 10, 142. A study has shown that surface functional modification such as antibodies or ligands targeting surface markers of senescent cells can significantly increase drug accumulation in tissues enriched with senescent cells such as adipose tissue and muscle while reducing exposure risk in non-target tissues 10. In addition, personalized biomarker-based delivery strategies, stimulus-responsive drug release systems, and receptor-mediated endocytosis pathways have also been extensively explored to further improve the precision and controllability of therapy. We anticipate that systematic combination of these approaches will markedly improve the targeting efficacy of senolytics, minimize off-target side effects, and offer a more practical technical route toward clinical translation.

Collectively, senescent cells play complex roles in the physiological and pathological processes of the musculoskeletal system. However, the specific mechanisms by which senescent cells act in different contexts, including their beneficial effects during acute muscle injury repair and harmful effects during chronic accumulation, still require further investigation. In addition, the development of current anti-aging therapeutic agents, such as senolytics, and targeted delivery strategies for musculoskeletal diseases remains at an early stage, and their specificity, safety, and long-term efficacy require systematic evaluation. With advances in single-cell technologies, novel carrier design, and precision medicine, more efficient and tissue-specific senescent cell-targeted therapies are expected to be developed in the future, providing new strategies for delaying the progression of and ameliorating musculoskeletal dysfunction.

## Figures and Tables

**Table 1:** Dual roles of cellular senescence in skeletal muscle injury and regeneration

Classification	Injury models	Types of senescent cells	Function	Ref.
Beneficial role	Acute skeletal muscle injury	Macrophages and FAPs	Transient emergence of the senescent phenotype promotes regeneration	67
	Exercise-induced muscle damage	FAPs	Establish a regenerative inflammatory state that promotes muscle regeneration	68
	Acute and chronic muscle injury	SA-β-gal-positive senescent cells	The favorable paracrine influence of injury-triggered senescence on cellular plasticity, further enhancing* in vivo* reprogramming	69
	Limb amputation of newt	Exogenously derived senescent cells	Facilitate dedifferentiation of mature muscle tissue to give rise to regenerative progenitors	70
Detrimental role	Skeletal muscle injury in geriatric mice	MuSCs	Impairs skeletal muscle regeneration	71
	Damaged muscles of young and old mice	MuSCs, macrophages and FAPs	Arrests stem cell proliferation and regeneration	21
	The tibialis anterior muscles of old mice were then injected with BaCl_2_	SA-β-gal positive cells	Insufficient muscle regenerative capacity	73
	Irradiation- and etoposide-induced senescence of primary FAPs* in vitro*	FAPs	Facilitates macrophage polarization toward the M2 phenotype and impairs skeletal muscle homeostasis	74

FAPs: fibro/adipogenic progenitors; SA-β-gal: senescence-associated beta-galactosidase; MuSCs: muscle stem cells.

**Table 2:** Characteristics of senescent cell and intervention strategies in different bone loss models

Disease model	*In vivo* study model	*In vitro* study model	Type of senescent cell	Key senescence markers	Intervention strategy	Outcome of the intervention	Ref.
Age-related bone loss/SOP	*Men1-*knockout mice and 24-month-old mice (aged) mice	Primary osteoblasts from *Men1*^flox/flox^ mice	Osteoblasts	p16^Ink4a^, SA-β-gal	Metformin treatment (senomorphic/mTORC1 inhibition)	Reduced osteoblast senescence & SASP; partially improved bone formation	120
	14-month-old mice	Aged human BMSCs	BMSCs	p16^Ink4a^, γH2AX, ROS, SA-β-gal	CXM102 (TFEB-mediated autophagy enhancer)	Alleviated age-related bone loss, reduced systemic inflammation	147
	SAMP6 mice	Primary mouse BMSCs (treated with AGEs-BSA)	BMSCs	p16^Ink4a^, p21^Cip1^, p53, H3K9me3, γH2AX, SA-β-gal	rAAV-Sirt3 overexpression, mitophagy activator (CCCP)	Activated mitophagy, alleviated BMSCs senescence, and attenuated SOP	101
	Naturally aged mice and Doxorubicin-induced aging model	Primary BMSCs (senescence induced by H_2_O_2_ and D-gal)	BMSCs	p16^Ink4a^, p21^Cip1^, SA-β-gal, γH2AX	Bone-targeted liposomes: (DSS)₆-liposomes loaded with quercetin	Eliminated senescent cells, restored BMSC function, and significantly increased bone formation	148
	20-month-old C57BL/6 mice	Primary BMSCs from aged mice	LepR+ BMSCs	p16^Ink4a^, p21^Cip1^, SA-β-gal	Local delivery of tetramethylpyrazine	Eliminated senescent MSPCs, attenuated trabecular bone loss	109
	20-24-month-old *INK-ATTAC* transgenic mice and C57BL/6 mice	Osteocyte-enriched cells	Osteocytes, myeloid cells	p16^Ink4a^, SA-β-gal, SADS	①Genetic: AP20187 (*INK-ATTAC* caspase activation),②Pharmacological: D+Q,③JAK inhibitor (ruxolitinib)	Improved bone mass, bone microstructure, and bone strength in aged mice	98
	*p16-3MR* transgenic mice (12-24 months old)	Osteoclast progenitors	Osteoclast progenitors (myeloid lineage)	p16^Ink4a^, SASP factors (IL-1α, IL-6)	Ganciclovir (GCV) activation of *p16-3MR* transgene	Senescent osteoclast progenitors were successfully eliminated, whereas senescent osteocytes were not. No effect on the SOP	149
	Aged mice (18-month-old)	BMSCs from osteoporosis patients	BMSCs	SA-β-gal, H3K27me3	Melatonin treatment (NSD2-mediated chromatin remodeling)	Enhanced chromatin accessibility, and alleviated age-related bone loss	150
	Aged mice and *optn*-/- mice	BMSCs	BMSCs	p16^Ink4a^, p21^Cip1^, SA-β-gal	Reactivating OPTN or inhibiting FABP3	Rescued BMSCs senescence, restored bone-fat balance, and alleviated bone loss	107
	Aged mice (20-month-old)	MC3T3-E1 osteoblastic cells	Osteoblasts	p21^Cip1^, p16^Ink4a^, p53, γH2AX, SA-β-gal	BMP9 treatment (activates Smad1-Stat1-p21^Cip1^ axis)	Inhibited osteoblast senescence, promoted bone formation, and improved bone mass and strength	121
	-	MC3T3-E1 osteoblasts, human MSCs	Osteoblasts	p21^Cip1^, γH2AX, SASP, SA-β-gal	PADI2 overexpression or NF-κB inhibition	Blocking SASP	115
	*p16-LOX-ATTAC* and *p16-INK-ATTAC* model mice	BMSCs from* p16-LOX-ATTAC* mice or* p16-LOX-ATTAC* mice	Osteocytes	p16^Ink4a^, SA-β-gal, TAF	Local (osteocyte-specific) vs. systemic senolysis (AP20187 drug)	Local senolysis partially replicates systemic benefits, while systemic senolysis provides comprehensive protection	131
	SAMP6	MLO-Y4 osteocytes, MC3T3-E1 osteoblasts	Osteocytes	p16^Ink4a^, SA-β-gal, γH2AX	miR-494-3p mimic or PTEN siRNA in osteocytes	Rescued osteoblast differentiation and prevented age-related bone loss	127
	Aged C57BL/6 mice (18 months)	MLO-Y4 osteocytes (H_2_O_2_-induced senescence)	Osteocytes	IL-6, p53, p21^Cip1^, γH2AX	RAPA	Reduced SASP	128
	SAMP6 mice	BMSCs and HUVECs following approximately 12-15, 20, and 18 cell passages, respectively	BMSCs	SA-β-gal, p16^Ink4a^, SASP	TPE-Gal	Selectively cleared senescent cells, improved bone mass and microarchitecture	130
Radiation-induced bone loss	BALB/c mice (subjected to a single 2 Gy dose of X-ray irradiation)	MLO-Y4 osteocytes (2 Gy γ-rays-induced senescence)	Osteocytes	p16^Ink4a^, p21^Cip1^, SAHF, γH2AX	JAK1 inhibitor	Inhibition of SASP relieved the impaired osteogenic and adipogenic differentiation of BMSCs	103
	-	Primary BMSCs (^137^Cs gamma rays)	BMSCs	SA-β-gal, p53/p21^Cip1^, γH2AX, ROS	JAK1 inhibitor	Blocking SASP alleviates irradiation-induced bone loss	112
Chemotherapy-induced bone loss	Doxorubicin treatment after stabilization of OVX-induced bone loss	-	Bone resident cell	p16^Ink4a^, HMGB1, SASP, SA-β-gal	①Genetic: AP20187 (*INK-ATTAC* caspase activation), ②Pharmacological: Using p38MAPK inhibitor or MAPKAPK2 (MK2) inhibitor	Clearing senescent cells, inhibiting the SASP Pathway	102
GIO	C57BL/6, p16-3MR mice, p16-cKO mice (Methylprednisolone treatment)	Primary BMSCs differentiated into adipocytes/preadipocytes (Dexamethasone treatment)	BMAds	p16^Ink4a^, p18^Ink4c^, p19^Ink4d^, HMGB1, Lamin B1, SASP, SA-β-gal, SADS	Adipocyte-specific deletion of p16^INK4a^, *p16-3MR* mice + GCV, COX2 inhibitor, PPARγ antagonists, D+Q, Ruxolitinib	Prevented the initiation of BMAd senescence and subsequent secondary senescence in bone vasculature and osteoblasts, partially rescuing bone loss	135
	C57BL/6 mice (Dexamethasone treatment)	MC3T3-E1 cell line (Dexamethasone treatment)	Osteoblasts	p16^Ink4a^, p21^Cip1^, SASP, SA-β-gal	Using siRNA to silence Cmpk2 expression in osteoblasts	Attenuated senescence, improved mitochondrial function, enhanced osteoblast differentiation	119
PMO	OVX mice (Six-month-old female C57BL/6 mice)	BMSCs (H_2_O_2_-induced or natural aging)	BMSCs	p16^Ink4a^, p21^Cip1^, p53, SA-β-gal	LRRc17 knockout	Rejuvenated BMSCs, ameliorated bone loss	105
	OVX middle-aged rats	MSCs	MSCs	p16^Ink4a^, p53, SA-β-gal, SASP	D+Q	Systemically prevented bone loss and locally rejuvenated bone regeneration	151
DOP	mouse model of type 2 diabetes	LepR+ MSCs	LepR+ MSCs	p16^Ink4a^, p21^Cip1^, Ki67, SA-β-gal, TNF-α, IL-6	Scutellarin treatment (Ezh2-Nrf2 signaling axis activation)	Prevented diabetes-induced bone loss by reducing cellular senescence and SASP	104

MEN1: multiple endocrine neoplasia type 1; SASP: senescence-associated secretory phenotype; BMSCs: bone marrow mesenchymal stem cells; TFEB: transcription factor EB; SAMP: senescence-accelerated mouse prone; AGEs: advanced glycation end products; AAV: adeno-associated virus; CCCP: carbonyl cyanide m-chlorophenyl hydrazine; SOP: senile osteoporosis; D-gal: D-galactose; LepR+: leptin receptor-positive; MSPCs: mesenchymal stem and progenitor cells; SADS: senescence-associated distension of satellites; D+Q: dasatinib plus quercetin; NSD2: nuclear receptor binding SET domain protein 2; OPTN: optineurin; FABP3: fatty acid-binding protein 3; BMP9: bone morphogenetic protein 9; PADI2: peptidyl arginine deiminase 2; TAF: telomere-associated foci; PTEN: phosphatase and tensin homolog; IL-6: interleukin-6; RAPA: rapamycin; TPE-Gal: galactose-modified tetraphenylethylene prodrug; SAHF: senescence-associated heterochromatin foci; ROS: reactive oxygen species; OVX: ovariectomy; HMGB1: high-mobility group box 1; BMAds: bone-marrow adipocytes; GCV: ganciclovir; COX2: Cyclooxygenase 2; PPARγ: perspective on peroxisome proliferator-activated receptor γ; TNF-α: tumour Necrosis Factor alpha; PMO: postmenopausal osteoporosis; DOP: Diabetic osteoporosis.

**Table 3:** Senescence-targeted therapeutic strategies for fracture healing

Treatment strategies	Targets	Underlying mechanism	Outcome of the intervention	Ref.
D+Q	Senescent cells	Triggering apoptosis	Decreased senescence and accelerated fracture healing	159, 166, 167, 177
ApoE antagonist	Senescent chondrocyte	Rescue the osteogenic suppression caused by Piezo1 deletion	Accelerated cartilage-to-bone transformation and fracture healing	168
Anti-inflammatory drug	Senescent SSPCs	Suppress the activation of NF-κB	Reduced senescence and enhanced osteogenic function	169
Combined treatment with BMP2 and CSF1 antagonist	Senescent SSPCs	Inhibition of BMP2 and CSF1	Restored senescence and concurrently eliminated the inflammatory, pro-osteoclastic microenvironment	173
Grancalcin neutralization	Senescent SSPCs	-	Enhanced fracture healing	176
Local H2 release	Senescent cells	-	Supported bone defect regeneration	178
Quercetin	Local senescent cells	-	Accelerated the repair of bone defects	179
Losartan and Fisetin	Senescent BMSCs	-	Reverse age-related impairments in bone homeostasis and promote healing	183
Targeted genetic clearance of p21^Cip1^-positive cells	Senescent OCHs and neutrophils	p21^Cip1^	Accelerated fracture healing	181
Deletion of p16^Ink4a^	p16^Ink4a^-positive senescent cells	p16^Ink4a^	Accelerated fracture healing	182

D+Q: dasatinib plus quercetin; APOE: apolipoprotein E; SSPCs: skeletal stem/progenitor cells; BMP2: bone morphogenetic protein 2; CSF1: colony-stimulating factor 1;

**Table 4:** ncRNAs involved in chondrocyte senescence and OA progression

ncRNAs Type	Molecule	Expression in OA	Functional role	Target/Signaling axis	Ref.
miRNA	miR-33-5p	Upregulated	Promotes senescence & OA	SIRT6	236
	miR-99b-5p	Upregulated	Promotes senescence & OA	MFG-E8/NF-κB	237
	miR-29b-5p	Downregulated	Successful cartilage repair and chondrocyte rejuvenation	TET1/p16^Ink4a^/p21^Cip1^	238
	miR-26b-5p	Downregulated	Reverses destructive effects in OA	Asporin/Smad2	239
	miR-140	Downregulated	Alleviates senescence & OA	Senescence markers	240
	miR-653-5p	Downregulated	Alleviates senescence & OA	IL-6/JAK/STAT3	241
	miR-6779	Downregulated	Reducing chondrocyte senescence and ECM loss	XIAP	247
	miR-24	Downregulated	Alleviates cellular senescence to promote chondrogenesis	TAOK1	248
lncRNA	ELDR	Upregulated	Promotes senescence & OA	Hedgehog/IHH	242
	LINC00623	Downregulated	Alleviates senescence & OA	miR-101/HRAS	249
	AC006064.4-201	Downregulated	Alleviate senescence and regulate metabolism in chondrocytes	PTBP1/CDKN1B	243
circRNA	CircP4HA3	Upregulated	Promotes senescence & OA	miR-5001-5p/THBS2	244
	CircGNB1	Upregulated	Promotes OA progression by enhancing oxidative stress	miR-152-3p/RNF219/CAV1	245
sdRNA	sdRNA-D43	Upregulated	Inhibited mitophagy and facilitated chondrocyte senescence and the development of OA	NRF1 and WIPI2	246

ncRNA: non-coding RNA; OA: osteoarthritis; lncRNAs: long non-coding RNA; miRNAs: microRNAs; circRNAs: circular RNAs; sdRNAs: small-derived RNAs; ECM: extracellular matrix; SIRT6: sirtuin 6; MFG-E8: milk fat globule-epidermal growth factor factor 8; TET1: ten-eleven-translocation enzyme 1; IL-6: interleukin-6; XIAP: X-linked inhibitor of apoptosis protein; TAOK1: thousand-and-one amino acid kinase 1; IHH: Indian hedgehog; HRAS: harvey rat sarcoma viral oncogene homolog; PTBP1: Polypyrimidine tract-binding protein 1; THBS2: thrombospondin 2; CAV1: Caveolin-1; NRF1: nuclear respiratory factor 1; WIPI2: WD repeat domain, phosphoinositide interacting 2.

## Abbreviations

AAV: adeno-associated virus
ABT-263: navitoclax
ADAMTS: a disintegrin and metalloproteinase with thrombospondin motifs
AF: annulus fibrosus
AGEs: advanced glycation end products
AOPPs: advanced oxidation protein products
APOD/E: apolipoprotein D/E
ASIC1a: acid-sensing ion channel 1a
ATAC-seq: assay for transposase-accessible chromatin sequencing
BCL2: B-cell lymphoma-2
BMAds: bone-marrow adipocytes
BMAT: bone marrow adipose tissue
BMMs: bone marrow macrophages
BMP9: bone morphogenetic protein 9
BMSCs: bone marrow mesenchymal stem cells
CCCP: carbonyl cyanide m-chlorophenyl hydrazone
CCL: C-C motif chemokine ligand
CDKs: cyclin-dependent kinases
CEP: cartilaginous endplate
ceRNA: competing endogenous RNA
circRNAs: circular RNAs
CM: conditioned medium
CMT: cyclic mechanical tension
COX2: Cyclooxygenase 2
CSA: cross-sectional area
CSF1: colony-stimulating factor 1
CTX: cardiotoxin
D+Q: dasatinib plus quercetin
DFO: deferoxamine
D-gal: D-galactose
DHE: dehydrocostus lactone
DM1-Gal: β-galactose-modified maytansine
DMM: destabilization of the medial meniscus
DOP: diabetic osteoporosis
ECM: extracellular matrix
EDTA: ethylenediaminetetraacetic acid
ET-1: endothelin-1
EVs: extracellular vesicles
FABP3: fatty acid-binding protein 3
FAPs: fibro/adipogenic progenitors
FBXW7: F-box and WD repeat domain containing 7
FGF: fibroblast growth factor
GCV: ganciclovir
GIO: glucocorticoid-induced osteoporosis
GLRX3: glutathione peroxidase 3
GPX4: glutathione peroxidase 4
HDAC3: histone deacetylase 3
HGPS: Hutchinson-Gilford progeria syndrome
HMGB1: high-mobility group box 1
Hmgcs2: 3-hydroxy-3-methylglutaryl-CoA synthase 2
IBM: inclusion body myositis
ICMT: intermittent cyclic mechanical tension
IDD: intervertebral disc degeneration
IL-6: interleukin-6
LDCD: lysosome-dependent cell death
LEF1: lymphoid enhancer factor 1
LepR+: leptin receptor-positive
lncRNAs: long non-coding RNA
LNPs: lipid nanoparticles
lncRNAs: long non-coding RNA
LRRc17: leucine-rich repeat containing 17
m^6^A: N6-methyladenosine
M-CSF: macrophage colony-stimulating factor
MEN1: multiple endocrine neoplasia type 1
METTL3: methyltransferase-like 3
MFG-E8: milk fat globule-epidermal growth factor factor 8
miRNAs: microRNAs
MLO-Y4: osteocyte-like cells
MMPs: matrix metalloproteinases
MSPCs: mesenchymal stem and progenitor cells
MuSCs: muscle stem cells
NAD+: nicotinamide adenine dinucleotide
ncRNA: non-coding RNA
NICD: Notch intracellular domain
NNMT: nicotinamide N-methyltransferase
NP: nucleus pulposus
NPCs: Nucleus pulposus cells
NSAIDs: non-steroidal anti-inflammatory drugs
NSD2: nuclear receptor binding SET domain protein 2
OA: osteoarthritis
OCHs: osteochondroprogenitors
ONFH: osteonecrosis of the femoral head
OPN: osteopontin
OPTN: optineurin
OVX: ovariectomy
ox-LDL: oxidized low-density lipoprotein
PADI2: peptidyl arginine deiminase 2
PCNA: proliferating cell nuclear antigen
PDZK1: PDZ domain containing 1
PLCG1: phospholipase Cγ1
PMO: postmenopausal osteoporosis
PPARγ: perspective on peroxisome proliferator-activated receptor γ
PTEN: phosphatase and tensin homolog
PUM1/2: Pumilio 1/2
RANKL: receptor activator of nuclear factor-κB ligand
RAPA: rapamycin
RIO: radiation-induced osteoporosis
ROS: reactive oxygen species
RPL35: ribosomal protein L35
rRNA: ribosomal RNA
SADS: senescence-associated distension of satellites
SAHF: senescence-associated heterochromatin foci
SAMP: senescence-accelerated mouse prone
SASP: senescence-associated secretory phenotype
SA-β-gal: senescence-associated beta-galactosidase
scRNA-seq: single-cell RNA sequencing
sdRNAs: small-derived RNAs
SEPHS1: selenium phosphate synthetase 1
shRNA: short hairpin RNA
SIDs: senescence identities
SIRT: sirtuin
SOP: senile osteoporosis
SSPCs: skeletal stem/progenitor cells
TAF: telomere-associated foci
TET1: ten-eleven-translocation enzyme 1
TFEB: transcription factor EB
TGF: transforming growth factor
TIPE2: TNF-α-induced protein 8-like protein 2
TLR3: toll-like receptor 3
TNF-α: Tumour Necrosis Factor alpha
TPE-Gal: galactose-modified tetraphenylethylene prodrug
TRIM15: tripartite motif-containing protein 15
uPAR: urokinase-type plasminogen activator receptor
VDR: vitamin D receptor
WTAP: WT1 associated protein
XIAP: X-linked inhibitor of apoptosis protein
YAP: Yes-associated protein
